# Epsin Family Member 3 and Ribosome-Related Genes Are Associated with Late Metastasis in Estrogen Receptor-Positive Breast Cancer and Long-Term Survival in Non-Small Cell Lung Cancer Using a Genome-Wide Identification and Validation Strategy

**DOI:** 10.1371/journal.pone.0167585

**Published:** 2016-12-07

**Authors:** Birte Hellwig, Katrin Madjar, Karolina Edlund, Rosemarie Marchan, Cristina Cadenas, Anne-Sophie Heimes, Katrin Almstedt, Antje Lebrecht, Isabel Sicking, Marco J. Battista, Patrick Micke, Marcus Schmidt, Jan G. Hengstler, Jörg Rahnenführer

**Affiliations:** 1 Department of Statistics, TU Dortmund University, Dortmund, Germany; 2 Department of Obstetrics and Gynaecology, University Hospital, Mainz, Germany; 3 Leibniz Research Centre for Working Environment and Human Factors (IfADo) at TU Dortmund University, Dortmund, Germany; 4 Department of Immunology, Genetics and Pathology, Uppsala University, Uppsala, Sweden; University of Alabama at Birmingham, UNITED STATES

## Abstract

**Background:**

In breast cancer, gene signatures that predict the risk of metastasis after surgical tumor resection are mainly indicative of early events. The purpose of this study was to identify genes linked to metastatic recurrence more than three years after surgery.

**Methods:**

Affymetrix HG U133A and Plus 2.0 array datasets with information on metastasis-free, disease-free or overall survival were accessed via public repositories. Time restricted Cox regression models were used to identify genes associated with metastasis during or after the first three years post-surgery (early- and late-type genes). A sequential validation study design, with two non-adjuvantly treated discovery cohorts (n = 409) and one validation cohort (n = 169) was applied and identified genes were further evaluated in tamoxifen-treated breast cancer patients (n = 923), as well as in patients with non-small cell lung (n = 1779), colon (n = 893) and ovarian (n = 922) cancer.

**Results:**

Ten late- and 243 early-type genes were identified in adjuvantly untreated breast cancer. Adjustment to clinicopathological factors and an established proliferation-related signature markedly reduced the number of early-type genes to 16, whereas nine late-type genes still remained significant. These nine genes were associated with metastasis-free survival (MFS) also in a non-time restricted model, but not in the early period alone, stressing that their prognostic impact was primarily based on MFS more than three years after surgery. Four of the ten late-type genes, the ribosome-related factors *EIF4B*, *RPL5*, *RPL3*, and the tumor angiogenesis modifier *EPN3* were significantly associated with MFS in the late period also in a meta-analysis of tamoxifen-treated breast cancer cohorts. In contrast, only one late-type gene (*EPN3*) showed consistent survival associations in more than one cohort in the other cancer types, being associated with worse outcome in two non-small cell lung cancer cohorts. No late-type gene was validated in ovarian and colon cancer.

**Conclusions:**

Ribosome-related genes were associated with decreased risk of late metastasis in both adjuvantly untreated and tamoxifen-treated breast cancer patients. In contrast, high expression of epsin (EPN3) was associated with increased risk of late metastasis. This is of clinical relevance considering the well-understood role of epsins in tumor angiogenesis and the ongoing development of epsin antagonizing therapies.

## Introduction

Breast cancer survival time is closely linked to distant metastatic recurrence. The absence of metastasis within the first years after diagnosis and primary therapy generally indicates a good long-term prognosis, but late metastatic events do still occur more than five years after diagnosis, however with a slowly decreasing risk [1–3]. As breast cancer survival is closely linked to distant metastatic recurrence, accurate prediction of late metastasis is therefore of high clinical relevance. For one, a patient’s long-term distress would be alleviated if it was possible to predict a low probability of late metastatic recurrence. Moreover, while unnecessary treatment of patients with a low risk of late metastasis could be avoided, high-risk patients could benefit from extended adjuvant endocrine therapy [4].

Clinicopathological factors, such as positive nodal status, large tumor size and positive estrogen receptor (ER) status, have been linked to late metastasis [3, 5–11]. On the other hand, gene signatures, primarily based on genes involved in proliferation, were successful in particular for prediction of early metastatic events [12]. In a prospective study of estrogen receptor-positive, node-negative patients, treated with anastrozole or tamoxifen [6], both the Oncotype DX 21-gene recurrence score [13] and the IHC4 immunohistochemistry panel (ER, PR, HER2, and Ki67) [14] contributed little to the prediction of late distant disease recurrence, whereas the PAM50 signature-based risk-of-recurrence (ROR) score [15] was shown to provide predictive power for late recurrence independent of clinical parameters. Also the breast cancer index (BCI) assay component *HOXB13*:*IL17BR*, originally identified in patients treated with tamoxifen monotherapy by comparing the gene expression profiles of recurrent and non-recurrent tumors [16,17], has been shown to identify high-risk patients 5–10 years after diagnosis, independent of conventional factors [18,19].

A number of explorative studies using transcriptome-wide search strategies to identify signatures specifically associated with tumor dormancy and late metastatic recurrence have been performed in breast cancer [11,20,21]. However, no study comprehensively analyzed a large number of breast cancer cohorts to identify and validate single genes with prognostic power for late metastasis, including FDR adjustment for multiple testing of the large number of candidate genes. Furthermore, it is not clear, to what extent genes associated with late metastatic recurrence in breast cancer can be extrapolated to other cancer types.

Using publicly available gene expression microarray data and a discovery-validation set approach, the aim of this study was to identify genes associated with metastatic recurrence (i) during the first three years after surgery (‘early-type genes’) and (ii) in the time period three years after surgery and later (‘late-type genes’), i.e. in patients who were metastasis-free during the first three years after surgery. In contrast to previous studies that mainly comprised adjuvantly treated patient populations with mixed nodal-status, only node-negative patients that were untreated in the adjuvant setting were included to avoid the potential difficulty of differentiating between the spontaneous course of the disease and treatment response. Genes associated with late metastatic recurrence were in a second step further validated in the today clinically more relevant group of ER positive breast cancer patients treated with adjuvant tamoxifen. Finally, we investigated whether genes associated with late metastatic recurrence in breast cancer showed the same association in non-small cell lung, ovarian and colon cancer.

## Material and Methods

### Datasets and data preprocessing

**Fig 1:**
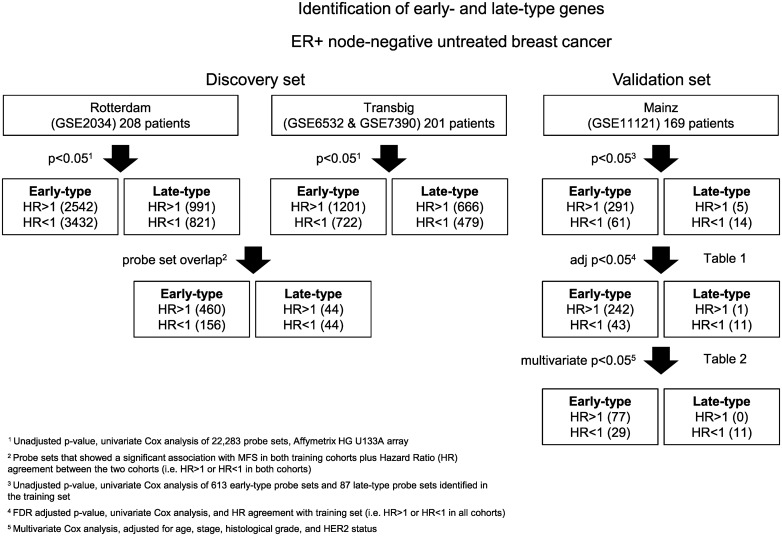
Identification of early- and late-type genes. Study design for identification and validation of early-type and late-type genes in node-negative, systemically untreated, ER-positive breast cancer. The number of probe sets identified in each step is given within parentheses.

Affymetrix GeneChip HG U133A gene expression microarray data, and a study design with two discovery sets and one validation set (Fig 1), was applied to identify late-type and early-type genes in ER positive, adjuvantly untreated, node-negative breast cancer. The Rotterdam (n = 208) (GSE2034) [22] and Transbig (n = 201) (GSE6532 and GSE7390) [23,12] datasets, accessed via the Gene Expression Omnibus (GEO) data repository [24], were used for gene discovery. Probe sets were defined as candidates if they were significantly associated with MFS in both cohorts (p<0.05) and showed hazard ratio agreement between the two cohorts (i.e. HR<1 or HR>1 in both cohorts). The association with late, or early, metastasis was then validated in the Mainz dataset (n = 169) (GSE11121) [25]. Frozen robust multiarray analysis (fRMA) [26] was used for normalization, since fRMA does make the expression values of different datasets more comparable. Clinicopathological characteristics for all patients, and for the subset of patients who did not develop a metastasis during the first three years after surgery, are summarized in S1A–S1F Table for all cohorts.

To extend the analysis to a currently clinically more relevant patient group (S1 Fig), seven datasets (GSE12093, GSE17705, GSE26971, GSE2990, GSE3494, GSE6532, GSE9195), including a total of 923 ER positive breast cancer patients treated with tamoxifen monotherapy in the adjuvant setting and with available information on MFS (793 patients when discarding observed times shorter than three years), were downloaded from GEO and manually curated. To further extend the analysis to other cancer types (S1 Fig), ten non-small cell lung cancer (NSCLC) datasets with information on overall survival (GSE14814, GSE19188, GSE29013, GSE30219, GSE31210, GSE3141, GSE37745, GSE4573, GSE50081, Shedden), totaling 1779 patients (1070 patients when discarding patients with observed times shorter than three years), eight ovarian cancer datasets with information on overall survival (GSE14764, GSE18520, GSE19829, GSE26193, GSE26712, GSE30161, GSE9891, Duke), totaling 922 patients (395 patients when discarding observed times shorter than 3 years), and four colon cancer datasets with information on disease-free survival (GSE14333, GSE17537, GSE39582, GSE41258), comprising 893 patients (486 patients when discarding patients with observed times shorter than three years) were downloaded from GEO and manually curated. For a summary of included datasets, see S1 Supporting Information.

### Statistical analysis

To identify early-type and late-type genes, given a fixed cutoff of c years, Cox models were fitted with adjusted times to metastasis. For the early-type analysis, times larger than c years were censored at c years, in order to neglect later events. For the late-type analysis, times smaller than c years were discarded, and from the remaining times c years were subtracted, in order to move the starting point to c years. For all cohorts and cancer types, the cutoff c = 3 years was used. For the node-negative untreated breast cancer cohorts, c = 5 was additionally used. Metastasis-free survival (MFS) was computed from the date of diagnosis to the date of distant metastasis. Patients who died of a non-tumor related cause were censored at the date of death. Univariate and multivariate Cox models adjusted for the available clinicopathological parameters were calculated using the R package ‘survival’ version 2.38 [27]. Survival rates were fitted with the Kaplan-Meier estimator. Survival functions were compared with the log-rank test.

ER and HER2 status were for the node-negative, untreated cohorts derived from the bimodally distributed mRNA levels of the corresponding genes (probe sets: *ESR1* 205225_at and *ERBB2* 216836_s_at) based on RMA normalized expression values, as stated in [28]. Categorization of clinical variables for the breast cancer cohorts was performed as follows: age: <50 vs. ≥50 years; grade: GI+GII vs. GIII; tumor size: ≤2cm vs. >2cm; HER2: negative vs. positive. For the NSCLC cohorts, clinical variables were categorized as follows: histology: squamous cell carcinoma vs. adenocarcinoma vs. large cell carcinoma vs. other; pTNM stage: I vs. II-IV; age: <70 vs. ≥70 years; sex: male vs. female; smoking status: never vs. current/ex-smoker. Clinical variables for the ovarian cancer cohorts were categorized as follows: age: <65 vs. ≥65 years; stage: I+II vs. III+IV; grade: GI+GII vs. GIII; histology: serous vs. other. The cutoff for the dichotomization of age was retrieved from [29], the corresponding publication to one of the included ovarian cancer cohorts. Categorization of clinical covariables for the colon cancer cohorts was performed as follows: age: <67 vs. ≥67 years; sex: male vs. female; grade: GI+GII vs. GIII; stage: 0+I+II vs. III+IV; tumor localization: distal vs. proximal vs. rectum. The cutoff for age was here determined by the median over all colon cancer cohorts.

Meta-analysis was performed with random effects models based on parameter estimates of log hazard ratios in univariate Cox models and corresponding standard errors. For combining single estimates into one pooled estimate inverse variance weighting was used. Results were visualized with forest plots, in which parameter estimates of all single studies and the pooled estimates along with their confidence intervals are plotted on top of each other. All analyses were performed using R version 3.2.1 [30] and the R package ‘meta’ version 4.3–0 [31].

## Results

### Identification of late-type genes

**Fig 2:**
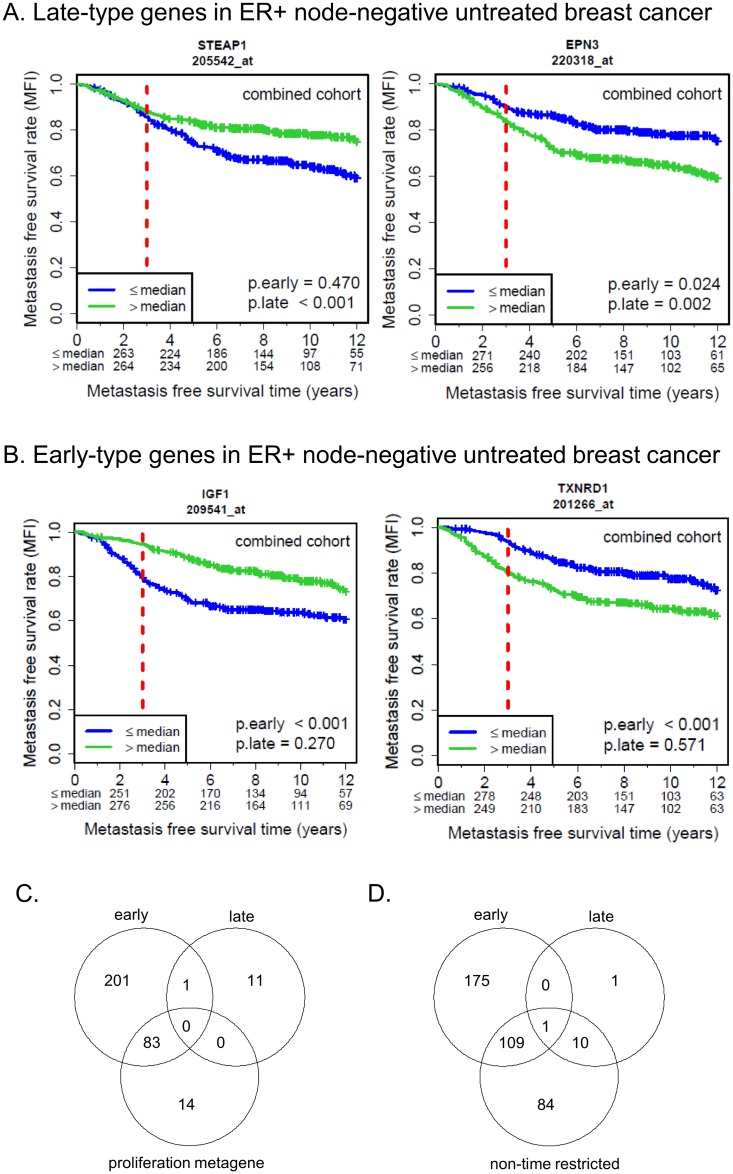
Validated early- and late-type genes. Kaplan-Meier plots representing validated late-type (A) and early-type (B) genes, for each showing one examples of one gene associated with better prognosis and one gene associated with worse prognosis. The median was used to differentiate between patients with low and high expression. Overlap between early-type and late-type genes with the previously described proliferation metagene (C). Overlap between early-type and late-type genes with genes associated with MFS in a conventional Cox model that considers the entire follow-up period (‘non-time restricted’) (D).

The analysis pipeline and a stepwise summary of the results are shown in Fig 1. Eighty-eight probe sets (81 genes) were significantly associated with late metastasis in both discovery cohorts, with a hazard ratio that was either increased or decreased in both cohorts (hazard ratio agreement) (S2A–S2C Table). In the second step, the association with late metastasis could be validated for 19 probe sets (16 genes) in the Mainz cohort, of which 12 (10 genes) were significant after FDR adjustment and showed hazard ratio agreement between all three cohorts (Table 1) (S2D Table). Examples of Kaplan-Meier plots for validated late-type genes are presented in Fig 2A.

**Table 1:** Late-type genes that predict metastasis-free survival three years after primary treatment and later.

Affy ID	Gene symbol	Rotterdam cohort		Transbig cohort		Mainz cohort		
		HR	p	HR	p	HR	p	p (fdr)
200081_s_at	RPS6	0.32	0.010	0.30	0.017	0.09	0.005	0.037
200715_x_at	RPL13A	0.20	0.011	0.38	0.035	0.15	0.003	0.037
200725_x_at	RPL10	0.08	0.003	0.09	0.026	0.03	0.007	0.044
200858_s_at	RPS8	0.17	0.004	0.17	0.004	0.07	0.007	0.044
200937_s_at	RPL5	0.34	0.012	0.28	0.001	0.15	0.003	0.037
205542_at	STEAP1	0.74	0.046	0.68	0.013	0.51	0.004	0.037
209134_s_at	RPS6	0.27	0.045	0.11	0.009	0.03	0.005	0.037
211073_x_at	RPL3	0.10	0.001	0.19	0.046	0.02	0.002	0.037
211938_at	EIF4B	0.26	0.007	0.25	0.002	0.13	0.003	0.037
215963_x_at	RPL3	0.10	0.001	0.17	0.019	0.04	0.001	0.037
217877_s_at	GPBP1L1	0.31	0.029	0.24	0.023	0.03	<0.001	0.030
220318_at	EPN3	2.31	0.036	2.07	0.024	2.56	0.005	0.037

Genes significant in the Rotterdam and Transbig cohorts were validated in the Mainz cohort (Fig 1). HR: hazard ratio; p: p-value; p (fdr): p-value after false discovery rate correction.

In the next step, the validated late type genes were adjusted to age, stage, grade, and HER2 status. Eleven probe sets (9 genes) remained significant in the multivariate Cox analysis (Table 2), indicating that most late-type genes are associated with time to metastasis independent of clinical parameters. Previously, proliferation, estrogen receptor and immune (T- and B-cell) metagenes were described and shown to represent biological motifs with a strong impact on breast cancer prognosis [25]. Therefore, the analysis was in addition adjusted to the proliferation metagene, estrogen receptor metagene and immune cell metagenes. The multivariate analysis demonstrated that 11 of 12 late-type probe sets (9 genes) were still independently associated with MFS after adjustment to the clinical factors and additionally to the metagenes (Table 2).

**Table 2:** Multivariate analysis of the ten late-type probe sets in the Mainz cohort.

		3 year cutpoint: multivariate analysis adjusted to			
		clinical factors		clinical factors + metagenes	
Affy ID	Gene symbol	HR	p	HR	p
200081_s_at	RPS6	0.10	0.015	0.10	0.031
200715_x_at	RPL13A	0.12	0.005	0.11	0.009
200725_x_at	RPL10	0.04	0.036	0.03	0.028
200858_s_at	RPS8	0.06	0.012	0.05	0.014
200937_s_at	RPL5	0.16	0.012	0.16	0.026
205542_at	STEAP1	0.54	0.013	0.56	0.022
209134_s_at	RPS6	0.04	0.024	0.03	0.036
211073_x_at	RPL3	0.03	0.008	0.01	0.003
211938_at	EIF4B	0.17	0.018	0.10	0.005
215963_x_at	RPL3	0.05	0.009	0.03	0.004
217877_s_at	GPBP1L1	0.04	0.001	0.02	<0.001
220318_at	EPN3	2.73	0.079	2.33	0.160

Validation of late-type probe sets. Multivariate analysis in the validation cohort (Mainz) adjusted to (i) clinical factors age, stage, grade and HER2 status (ii) age, stage, grade, HER2 status and additionally to the proliferation, estrogen receptor, B-cell and T cell-associated metagenes. HR: hazard ratio; p: p-value.

We then asked whether the identified late-type genes were associated with MFS also after five years, so a Cox model starting at five years after surgery was fitted, discarding event times shorter than five years and subtracting five years from the remaining times. All but one late-type probe set were significantly associated with MFS also after five years (S3 Table). Analysis of even later time periods was not feasible due to the small number of metastatic events.

Information about the functions of the validated late-type genes is briefly summarized in Table 3. Six of the ten late-type genes encode components of ribosomal 40S and 60S subunits (*RPL3*, *RPL5*, *RPL10*, *RPL13A*, *RPS6*, *RPS8*) and one encodes a binding partner of the 40S ribosomal subunit (*EIF4B*). The function of *GPBP1L1* is not well-known, but it has been reported to interact with eukaryotic translation initiation factor 3 (*EIF3B*). In addition to functioning as ribosomal components, ribosomal proteins have been implicated in for instance stress signaling, apoptosis, regulation of replicative life span, and translational silencing (Table 3). Epsin 3 (*EPN3*), reported to mediate epithelial cell migration, was the only validated late-type gene associated with shorter MFS.

**Table 3:** Summary of known biological functions for the validated late-type genes.

Gene symbol	Gene name	Function
RPL3	Ribosomal Protein L3	Component of the 60S ribosomal subunit; RPL3-mediated p21 upregulation induces G₁/S cell cycle arrest or apoptosis in the absence of p53 [37]; Essential for response to 5-FU and oxaliplatin and involved in DNA repair [43]
RPL5	Ribosomal Protein L5	Component of the 60S ribosomal subunit; Involved in p53 stress signaling [40,41]; Upon stress, RPL5 binds to MDM2, thereby activating p53 [44]; Found to be mutated in glioblastoma and T-cell acute lymphoblastic leukemia (T-ALL), as well as in Diamond Blackfan anemia (DBA), a ribosomopathy connected with an increased lifetime risk of cancer [45]
RPL10	Ribosomal Protein L10	Component of the 60S ribosomal subunit; Involved in replicative life span regulation in yeast [42]; Mutated in a subset of T-ALL patients [46]
RPL13A	Ribosomal Protein L13a	Component of the 60S ribosomal subunit; Part of the IFN-gamma-activated inhibitor of translation (GAIT) complex [47]; Involved in translational silencing [48]
RPS6	Ribosomal Protein S6	Component of the 40S ribosomal subunit; Promotes lipogenesis via the AKT—mTORC1–RPS6 pathway [38]; A mediator of mTOR-inhibitor anti-tumoral activity in renal cell cancer [49]; Hypoxia inhibits translation by suppression of RPS6, independent of HIF [39]
RPS8	Ribosomal Protein S8	Component of the 40S ribosomal subunit; Interacting partner of CDK11p46, regulates translation and sensitizes cells to Fas ligand-induced apoptosis [36]
EIF4B	Eukaryotic Translation Initiation Factor 4B	Required for the binding of mRNA to ribosomes; Phosphorylated and activated by Ras-MAPK and PI3K-mTOR; depletion is linked to lower proliferation and promotion of apoptosis [50]
GPBP1L1	GC-Rich Promoter Binding Protein 1-Like 1	Function unknown; Possible transcription factor; Experimental evidence (two hybrid assay) for interaction with eukaryotic translation initiation factor 3 (EIF3B) [51]
STEAP1	Six Transmembrane Epithelial Antigen Of The Prostate 1	Metalloreductase; Upregulated in cancers and a possible target for immunotherapy [52]; Correlates with tumor grade and inversely with estrogen receptor immunoreactivity and tumor in breast cancer [53]
EPN3	Epsin 3	Endocytic adaptor; Overexpression of epsins induces migration [54,55]

Official gene symbol, gene name and summary of known biological functions for validated late-type genes, based on information from Entrez Gene, UniProt and literature.

While not the main focus of this investigation, it is also worth noting that when the entire analysis pipeline described above was repeated for ER negative breast cancers (Rotterdam n = 78; Transbig n = 79; Mainz n = 31), no late-type gene was identified (data not shown), probably due to power restrictions because of low case numbers and few late events (Rotterdam n = 52; 2 events, Transbig n = 62; 10 events, Mainz n = 24; 5 events).

### Identification of early-type genes

The same analysis pipeline was then applied to identify early-type genes (Fig 1). 616 probe sets (494 genes) were significantly associated with early metastasis in both discovery cohorts and showed hazard ratio agreement between the two discovery cohorts (S2A–S2C Table). The association with early metastasis could be validated for 352 probe sets (291 genes) in the Mainz cohort, of which 285 (243 genes) were significant also after FDR adjustment and showed hazard ratio agreement between all three cohorts (S2D Table). Examples of Kaplan-Meier plots for validated early-type genes are presented in Fig 2B. Of the 285 validated probe sets, 106 (87 genes) remained significant after adjustment to age, stage, grade, and HER2 status (S4 Table). After additional adjustment to the proliferation, estrogen receptor and immune cell metagenes previously identified by [25], only 17 probe sets (16 genes) remained significant (S4 Table). A relatively large fraction of the 285 validated early-type probe sets overlapped with the probe sets included in the proliferation metagene (Fig 2C). The proliferation metagene has been shown to be associated with worse prognosis in a non-time restricted model [25] and most of these genes are also associated with early metastasis in the here performed Cox model restricted to the first three years after surgery.

### Overlap of late- and early-type genes with genes identified by a non-time restricted model

The separate analysis of late-type and early-type genes indicated that these genes upon first sight appeared to belong to different categories: a small number of late-type genes, mostly associated with longer MFS, and a much larger number of early-type genes, primarily associated with proliferation and mostly associated with shorter MFS. To further understand whether late-type genes are associated with MFS exclusively in the period three years after surgery and later, correspondingly, if early-type genes are associated with MFS exclusively in the first three years after surgery, the overlap with genes associated with MFS in a non-time restricted model was determined. When the same analysis pipeline was applied to identify genes associated with metastatic recurrence using a non-time restricted model, 626 probe sets (519 genes) were significantly associated with metastasis in both discovery cohorts and showed hazard ratio agreement between the two discovery cohorts (S2A–S2C Table). The association with metastasis could be validated for 277 probe sets (233 genes) in the Mainz cohort, of which 204 (174 genes) were significant also after FDR adjustment and showed hazard ratio agreement between all three cohorts (S2D Table). Only one probe set (*EIF4B*) was found in the overlap of early- and late-type genes and genes significant in the non-time restricted model (Fig 2D). All but one late-type probe set (11 probe sets; 9 genes) and 38% of the early-type probe sets (110 probe sets; 100 genes) were also associated with metastatic recurrence considering the entire follow-up period (Fig 2D). One probe set was associated with MFS in the late period only, whereas 175 probe sets were associated with MFS in the early period only (Fig 2D).

Analyzing the identified late-type genes in the early and complete time period in each cohort separately, 17–75% (range of the three cohorts) of late-type genes were found to be associated with MFS also in the early period and 92–100% in the non-time restricted analysis (S5A–S5C Table). Correspondingly, 13–18% of early-type genes were found to be associated with MFS in the late period and 66–85% in the non-time restricted analysis (S6A–S6C Table). In conclusion, this complex scenario suggests a concept where prognostic genes are primarily influential during an early period after diagnosis, but major differences exist regarding to which degree their influence is maintained after three years and later. The majority of genes lost their prognostic influence after longer time periods (‘early-only genes’), whereas a small number of genes maintained their significance also after three years (late-type genes). One probe set (*STEAP1*) was associated with MFS in the late period only, whereas 175 probe sets were associated with MFS in the early period only (Fig 2D). While the existence of ‘early-only genes’ is unquestionable, we hesitate to claim the existence of ‘late-only genes’ based on one probe set only.

### Validation of late-type genes in ER positive breast cancer treated with adjuvant tamoxifen

**Fig 3:**
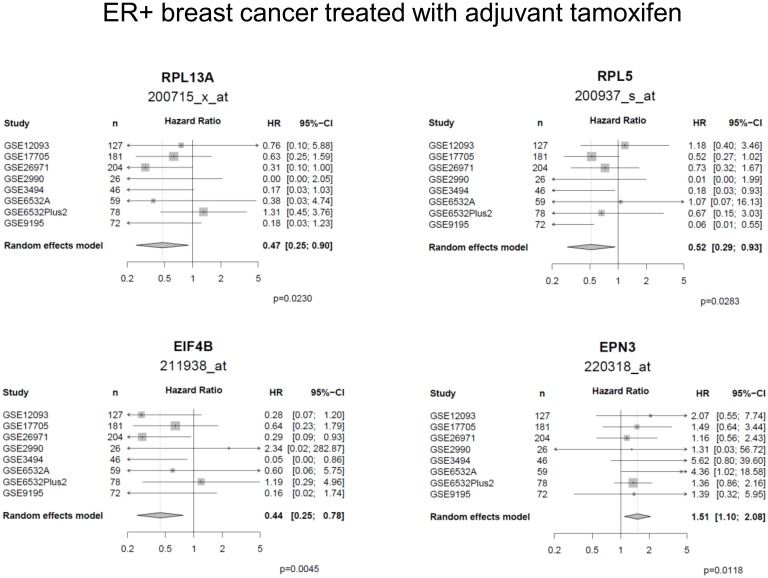
Validation of late-type probe sets in tamoxifen-treated cohorts. Forest plots for the late-type probe sets that were significantly associated with prognosis in the meta-analysis of ER-positive breast cancers treated with adjuvant tamoxifen.

We then tested if the identified late-type genes showed a prognostic impact in the time period three years after surgery also in a today more clinically relevant situation, as ER positive breast cancer patients currently often are treated with tamoxifen after surgical removal of the primary tumor. Eight cohorts, including a total of 923 ER positive patients treated with tamoxifen in the adjuvant setting, were analyzed. Discarding event times shorter than three years, 793 patients remained (S1 Fig). Three late-type genes, *EIF4B*, *RPL5* and *RPL3*, were found to be significantly associated with late metastasis in two or more cohorts (S7A Table), all associated with longer MFS in the univariate Cox analysis, in agreement with findings in the node-negative, untreated breast cancer cohorts. Including all cohorts in a meta-analysis, *EIF4B*, *EPN3*, *RPL13A* and *RPL5* were significantly associated with late metastasis and corresponding forest plots are visualized in Fig 3.

### Prognostic influence of late-type genes in non-small cell lung, ovarian and colon cancer

We then aimed to determine if late-type genes identified in breast cancer are relevant also in other cancer types. The association with overall survival (OS) or disease-free survival (DFS) in patients who were still alive or disease-free three years after primary surgery was therefore analyzed in non-small cell lung cancer (OS), ovarian cancer (OS) and colon cancer (DFS) (S1 Fig). Results are comprehensively reported for each cohort in S7B–S7D Table and briefly summarized below.

Ten NSCLC cohorts, including in total 1070 patients when discarding event times shorter than three years, were analyzed. One late-type gene (*EPN3*) was significantly associated with shorter OS, and two late-type genes (*RPL3*, *EIF4B*) with longer OS, in more than one cohort (S7B Table), in agreement with findings in breast cancer. Additionally, four late-type genes (*RPL13A*, *RPL5*, *RPL8*, *GPBP1L1*) showed a significant association with OS in one cohort only. In the analysis of eight ovarian cancer cohorts, including in total 395 patients when discarding event times shorter than three years, only one late-type gene (*RPS6*) showed a significant association with OS and only in one cohort (S7C Table), higher gene expression being associated with better outcome, in agreement with the observed association with longer MFS in breast cancer. Finally, in the analysis of four colon cancer cohorts, comprising 486 patients when discarding event times shorter than three years, only one gene (*RPL13A*) was significantly associated with longer DFS in one cohort (S7D Table), also here higher gene expression being associated with better outcome in agreement with the observed association with longer MFS in breast cancer.

Overall, findings in breast cancer were poorly reproducible in other cancer types. Furthermore, as evident from S7B–S7D Table, several late-type genes showed a significant, but opposite, association with prognosis in one or more cohorts compared to the initial analysis of breast cancer, exemplified by *RPL10*, *EIF4B*, *GPBP1L1* in NSCLC and *RPL3*, *RPS6* and *STEAP1* in ovarian cancer.

## Discussion

Relatively little is still known about factors that promote, or protect against, late-occurring distant metastasis in breast cancer and there is a need to further outline if the expression patterns of specific genes are linked to early and late metastatic recurrence. In this study, genes associated with early and late metastasis were identified in a transcriptome-wide manner based on time restricted Cox regression models and a sequential validation approach. Gene identification and validation was performed using publicly available gene expression microarray data from node-negative, adjuvantly untreated, breast cancer patients with positive ER status. Genes associated with late metastasis were then further evaluated in ER positive breast cancer patients treated with adjuvant tamoxifen. The adjuvant untreated cohort will harvest genes associated with the spontaneous progression of breast cancer, while the tamoxifen-treated cohort will additionally lead to identification of genes associated with response to tamoxifen. Nevertheless, this sequential rationale is justified, since the discovery cohort of untreated patients reduces the number of genes for validation in the tamoxifen-treated cohort and thereby ameliorates the multiple testing problem. Since validation is performed in the tamoxifen-treated cohort, the confirmed genes are of clinical relevance, because anti-estrogenic therapy of breast cancer represents a clinical standard. Moreover, the analysis was extended to non-small cell lung, ovarian and colon cancer to determine if the prognostic impact could be validated across diverse cancer types.

Ten late-type and 243 early-type genes were identified. After adjustment to clinicopathological factors and previously established proliferation- and immune-related signatures, all but one late-type gene was independently associated with MFS while the number of significant early-type genes was reduced to 16. Late-type genes were previously reported to play a role in processes such as apoptosis, stress signaling, and hypoxia (Table 3), and higher expression was associated with reduced risk of late metastasis, with one exception (*EPN3*). Early-type genes were on the other hand dominated by proliferation-associated factors. However, the indication that different gene sets might be related to early and late metastasis must be interpreted in light of the large overlap between both late- and early-type genes with genes identified using a non-time restricted model. In fact, with one exception (Fig 2D), all identified late-type genes were also associated with MFS in the non-time restricted model, in agreement with the concept that no additional metastasis-associated genes are identified when restricting the analysis to the patient population who did not experience metastatic recurrence during the first three years after surgery.

The difficulties in predicting long-term breast cancer prognosis are underscored by the fact that only ten late-type genes were significantly associated with MFS in the patient population that was metastasis-free for the first three years after surgical tumor resection. Also, other studies using a transcriptome-wide strategy to identify genes associated with late metastatic recurrence in breast cancer have yielded different lists of candidate genes [11,20,21]. This could be due to different statistical approaches, analysis of dissimilar patient populations, or a combination of both. From a methodological perspective, any high-dimensional analysis, such as that of transcriptome-wide gene expression data, requires a strategy that controls the number of false positive discoveries. One strategy to address the problem of multiple testing is sequential validation, where significant genes identified in a discovery set enter as candidates in a validation set. We previously recommended an optimized order for such a stepwise procedure, where the datasets with the largest sample size (and the lowest measurement variance) are used for discovery steps and the datasets with the smallest sample size for validation steps [32]. Based on this approach, the Rotterdam and Transbig cohorts were here used for gene discovery and the Mainz cohort for validation. As an alternative, a meta-analysis was performed for the three untreated, node-negative breast cancer cohorts and all probe sets on the Affymetrix HG U133A array to first consider the association in each cohort independently and then combine them into one measure. Also with this approach, the ten late-type genes were identified (S8 Table) and the overlap between the three cohorts for probe sets associated with MFS in the early and late time period is illustrated in S2 Fig.

One limitation of the present study was the number of metastatic events in long-term breast cancer survivors. Due to a low number of events, late metastasis could only be analyzed in the time period beyond three and five years after primary diagnosis and not for an even later time period (17 events after ten years, as compared to 98 and 53 after three and five years, respectively, and hence insufficient statistical power). The fact that more early-type genes (n = 243) were identified compared to the number of late-type genes (n = 10) can, however, not be explained by statistical power, as a similar number of metastatic events occurred within the first three years after surgery (n = 74) compared to the time period beyond three years (n = 98). However, it is perhaps not surprising that most genes associated with MFS identified in the analysis of primary tumor tissue predict early metastasis, since the probability of the tumor acquiring additional mutations, and thereby changes in metastatic capacity, increases with time during tumor evolution.

Previous studies focussed on associations of clinicopathological parameters with late metastasis [3,5,7–10]. Estrogen receptor status has long been discussed as a factor that influences the timing of metastatic recurrence [3,9,10]. However, based on a meta-analysis of our three untreated, node-negative cohorts, ESR1 expression was not significantly associated with late metastasis (p = 0.24). Focusing on genes involved in a pre-defined pathway, a 158-gene signature associated with c-Src activity, proposed by Zhang and colleagues, was shown to be associated with late-onset bone metastasis in breast cancer [33]. There was no overlap between the genes included in the c-Src signature and the ten late-type genes identified in our multi-cohort analysis pipeline. Extending the analysis to include also estrogen receptor-negative patients, in analogy to the Zhang et al. study cohort, and using a genome-wide meta-analysis approach, 1558 out of 22283 probe sets were associated with late metastasis (unadjusted p<0.05). No significant over-representation of late-type genes in the c-Src signature (14 out of 158 genes) compared to the genome-wide fraction of late-type genes was observed (p = 0.216, Fisher test) (S9 Table).

Recently, a study in 252 breast cancer patients has been published which aimed to identify genes predicting late distant metastasis in adjuvantly untreated, ER-positive, HER2 negative patients [11]. Considering the similarity of the study population and the common goal, it is surprising that we could not confirm a single gene of the 241-gene signature for late distant metastasis of Mittempergher and colleagues. Upon thorough analysis and application of the statistical methods of [11] to our patient cohorts we found that the different statistical approach explains the discrepancy: As additional inclusion criteria, Mittempergher et al. required HER2 negativity and MammaPrint low-risk status, based on the assumption that processes unique to late metastatic events are likely to be different from those captured by predictors of high risk of early recurrence, such as MammaPrint. Prediction Analysis for Microarrays (PAM) identified 241 probe sets (corresponding to 230 genes) predictive of late metastasis comparing the late relapse group (5–15 years) with a control group who did not experience disease recurrence during ten years of follow-up. There was no overlap between these 230 late distant metastasis associated genes and our ten late-type genes, while three of the 230 genes were among our identified early-type genes. Additionally, the authors evaluated the association to late metastasis for individual genes with time to event as a continuous variable. For this analysis the expression values of each probe set were dichotomized using the median as cutpoint. To evaluate if the hazard ratios in the corresponding patient groups differed, the authors used a test that puts more weight on late differences (Fleming and Harrington’s G-rho test with rho = -1). This test weights differences between hazard rates with the inverse of the Kaplan-Meier estimate at every time point. Based on this procedure, two genes were identified: cholesterol 25-hydrolase (*CH25H*) and follistatin-like 4 (*FSTL4*), the first of which was validated in three publicly available data sets (non-overlapping with the datasets analyzed in our study). Neither *CH25H* nor *FSTL4* were among the late-type genes identified by our analysis strategy. Using the statistical methods of [11] in our analysis pipeline, two other genes were identified: FtsJ RNA methyl transferase homolog 2 (*FTSJ2*) and epidermal growth factor receptor pathway substrate 15-like 1 (*EPS15L1*). Two methodical reasons explain this discrepancy. The PAM analysis requires a dichotomization of the disease recurrence time which implies an information loss compared to Cox regression analysis. Furthermore, the additional weight for late differences in the G-rho test depends heavily on the proportion of censored observations. If the proportion of late-censored observations is high, this approach is similar to an unweighted log-rank test. However, when only few late censored times are available, only very late events are considered for testing differences. In contrast, the approach of our study either focusses on events during the first three years, ignoring later events, or ignores the first three years and considers all events thereafter. This relatively simple approach is robust, allows differentiation of early and late type genes and avoids the bias that may be caused by censored observations in the G-rho test.

Extending our analysis to breast cancer patients adjuvantly treated with tamoxifen, four late-type genes (*EIF4B*, *RPL5*, *RPL3*, *EPN3*), identified using our sequential validation strategy, were validated to be significantly associated with MFS in a meta-analysis including eight tamoxifen-treated breast cancer cohorts, when restricting the analysis to patients who were metastasis-free during the first three years after surgery. In contrast, few late-type genes showed consistent survival associations in other cancer types, with the exception of epsin 3 (*EPN3*), which was associated with late metastatic recurrence in adjuvantly untreated and tamoxifen-treated breast cancer as well as with worse outcome in the period three years after surgery and later in two non-small cell lung cancer cohorts. EPN3 belongs to the epsin family of endocytic adaptor proteins, originally described to be involved in clathrin-mediated internalization of cell surface receptors and lately reported to play a role in angiogenesis as well as tumor cell migration and invasion [34]. Interestingly, endothelium-specific epsin deficiency has been shown to block tumor progression in murine models by disrupting tumor angiogenesis. Epsins recognize ubiquitinated VEGFR2, support its degradation and reduce VEGF signaling. As a therapeutic strategy, a synthetic peptide that blocks epsin-VEGFR2 interactions has been shown to result in dysfunctional vasculature unable to support the growing tumor [35]. The present study reports for the first time that expression of a member of the epsin family is associated with poor outcome in two common human cancers.

While the role of epsin in tumor progression has already been established, the association of ribosome-related genes with better prognosis remains difficult to understand. Two of the ribosomal proteins, RPS8 and RPL3 have been reported to be involved in modulation of apoptosis sensitivity [36,37]. RPS6 and RPL5 are involved in stress signaling or hypoxia response [38–41]. RPL10 has been shown to be involved in replicative life span regulation [42]. Whether these mechanisms are sufficient to explain the here observed role of ribosomal proteins in late metastasis remains to be studied.

In summary, we presented a comprehensive study of breast cancer cohorts regarding identification of single genes with prognostic power for late metastasis. We rigorously adjusted for multiple testing of the large number of candidate genes with the FDR (false discovery rate) approach. Ten genes were significantly associated with MFS in the patient population that was metastasis-free during the first three years after tumor resection. This was true also after adjustment for clinicopathological parameters. We identified ribosomal proteins associated with better prognosis. Their role in suppression of metastasis remains to be elucidated. On the other hand high expression of epsin (*EPN3*) with its well-established role in tumor angiogenesis, conferring worse prognosis in breast and lung cancer, is of high clinical relevance considering the ongoing development of epsin antagonizing therapies.

## Supporting Information

S1 Fig Number of observations in the validation datasets. Genes associated with late metastatic recurrence were further validated in ER-positive patients treated with adjuvant tamoxifen and investigated in non-small cell lung, ovarian and colon cancer.
(PPTX)

S2 Fig Venn diagrams for the number of probe sets significantly associated with MFS in the early and late period. (PPTX)

S3 Fig Correlation between late-type genes and clinical parameters. Beanplots of expression values of the validated late-type genes in association with age, stage, histological grade and HER2 status.
(PDF)

S1 Supporting Information Summary over all analyzed datasets (ER-positive, tamoxifen-treated breast cancer, non-small cell lung cancer, ovarian cancer and colon cancer) and the data curation process. (DOCX)

S1 Table Summary of clinicopathological characteristics of the node-negative, untreated breast cancer cohorts. Clinicopathological characteristics for all patients and for the subset of ER-positive patients included in the late analysis for the Mainz (A-B), Rotterdam (C-D), and Transbig (E-F) cohort.
(DOCX)

S2 Table Identified late- and early-type genes. Late- and early-type genes identified in the Rotterdam cohort (A), Transbig cohort (B), overlap of Rotterdam and Transbig cohorts (C), and validated in the Mainz cohort (D). Additionally, results of a conventional Cox model without time restriction are shown (A-D). Affymetrix probe set IDs and official gene symbols are given. HR: hazard ratio; p: p-value unadjusted; fdr: false discovery rate adjusted.
(XLSX)

S3 Table Univariate analysis of the late-type probe sets in the period beyond five years after surgery in the validation cohort (Mainz). (DOCX)

S4 Table Validated early-type genes. Multivariate analysis of the validation cohort (Mainz) adjusted to age, stage, grade and HER2 status, and additionally to the proliferation, estrogen receptor, B-cell and T-cell associated metagenes. Affymetrix probe set ID numbers and official gene symbols are given. HR: hazard ratio; p: p-value; fdr: false discovery rate adjusted.
(XLSX)

S5 Table Summary of the ten late-type genes in the node-negative untreated cohorts. Summary of the ten late-type genes in the Mainz (A), Rotterdam (B) and Transbig (C) cohort, with results of the univariate Cox analysis are shown for both the early (up to three years) and the late period (from three years), as well as for a conventional model without time restriction. HR: hazard ratio; CI: confidence interval.
(XLSX)

S6 Table Summary of the 243 early-type genes in the node-negative untreated cohorts. Summary of the 243 early-type genes in the Mainz (A), Rotterdam (B) and Transbig (C) cohort, with results of the univariate Cox analysis are shown for both the early (up to three years) and the late period (from three years), as well as for a conventional model without time restriction. HR: hazard ratio; CI: confidence interval.
(XLSX)

S7 Table Validation of the ten late-type genes in other cancer datasets. Validation of the ten late-type genes in eight ER-positive breast cancer patients treated with adjuvant tamoxifen monotherapy (A), in ten non-small cell lung cancer cohorts (B), in eight ovarian cancer cohorts (C), and in four colon cancer cohorts (D). Results of the univariate and multivariate Cox analysis, adjusted for the available clinical covariables in each cohort, for the late period (from three years) are shown. HR: hazard ratio; CI: confidence interval.
(XLSX)

S8 Table Validated late-type genes that predict metastasis-free survival three years after primary treatment and later in the three node-negative, untreated breast cancer cohorts and in a meta-analysis of all three cohorts. (DOCX)

S9 Table c-Src associated genes. Genes from the c-Src signature identified by Zhang et al. (2009) were analyzed in the cohort of 766 node-negative breast cancer patients using a meta-analysis approach with random effects to identify late-type genes. Significant genes (p<0.05) are indicated by red color. HR: hazard ratio; raw p: p-value without adjustment for multiple testing; adj. p: p-value adjusted for multiple testing; rank: rank of probe set according to p-value.
(XLSX)
