# Supplementary material for: Epsin Family Member 3 and Ribosome-Related Genes Are Associated with Late Metastasis in Estrogen Receptor-Positive Breast Cancer and Long-Term Survival in Non-Small Cell Lung Cancer Using a Genome-Wide Identification and Validation Strategy
Source: PLoS One. 2016 Dec 7;11(12):e0167585. doi: 10.1371/journal.pone.0167585 (PMC5142791; doi:10.1371/journal.pone.0167585)
Supplement: S1 Fig — Genes associated with late metastatic recurrence were further validated in ER-positive patients treated with adjuvant tamoxifen and investigated in non-small cell lung, ovarian and colon cancer. (PPTX) [file pone.0167585.s001.pptx]

## Slide 1
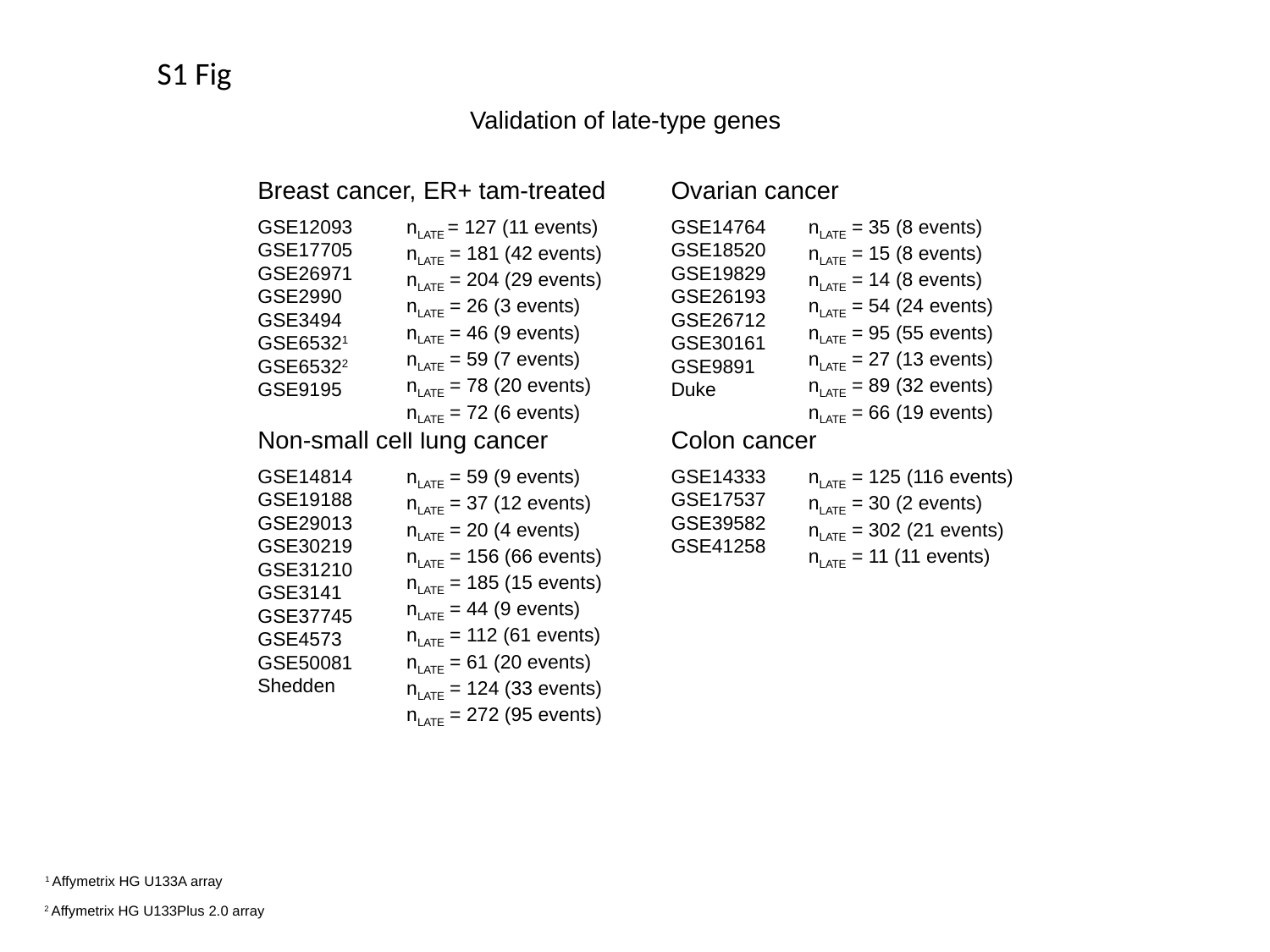

S1 Fig
Validation of late-type genes
Breast cancer, ER+ tam-treated
Ovarian cancer
GSE12093
GSE17705
GSE26971
GSE2990
GSE3494
GSE65321
GSE65322
GSE9195
nLATE = 127 (11 events)
nLATE = 181 (42 events)
nLATE = 204 (29 events)
nLATE = 26 (3 events)
nLATE = 46 (9 events)
nLATE = 59 (7 events)
nLATE = 78 (20 events)
nLATE = 72 (6 events)
GSE14764
GSE18520
GSE19829
GSE26193
GSE26712
GSE30161
GSE9891
Duke
nLATE = 35 (8 events)
nLATE = 15 (8 events)
nLATE = 14 (8 events)
nLATE = 54 (24 events)
nLATE = 95 (55 events)
nLATE = 27 (13 events)
nLATE = 89 (32 events)
nLATE = 66 (19 events)
Non-small cell lung cancer
Colon cancer
GSE14814
GSE19188
GSE29013
GSE30219
GSE31210
GSE3141
GSE37745
GSE4573
GSE50081
Shedden
nLATE = 59 (9 events)
nLATE = 37 (12 events)
nLATE = 20 (4 events)
nLATE = 156 (66 events)
nLATE = 185 (15 events)
nLATE = 44 (9 events)
nLATE = 112 (61 events)
nLATE = 61 (20 events)
nLATE = 124 (33 events)
nLATE = 272 (95 events)
GSE14333
GSE17537
GSE39582
GSE41258
nLATE = 125 (116 events)
nLATE = 30 (2 events)
nLATE = 302 (21 events)
nLATE = 11 (11 events)
1 Affymetrix HG U133A array
2 Affymetrix HG U133Plus 2.0 array
