# Supplementary material for: Epsin Family Member 3 and Ribosome-Related Genes Are Associated with Late Metastasis in Estrogen Receptor-Positive Breast Cancer and Long-Term Survival in Non-Small Cell Lung Cancer Using a Genome-Wide Identification and Validation Strategy
Source: PLoS One. 2016 Dec 7;11(12):e0167585. doi: 10.1371/journal.pone.0167585 (PMC5142791; doi:10.1371/journal.pone.0167585)
Supplement: S2 Fig — (PPTX) [file pone.0167585.s002.pptx]

## Slide 1
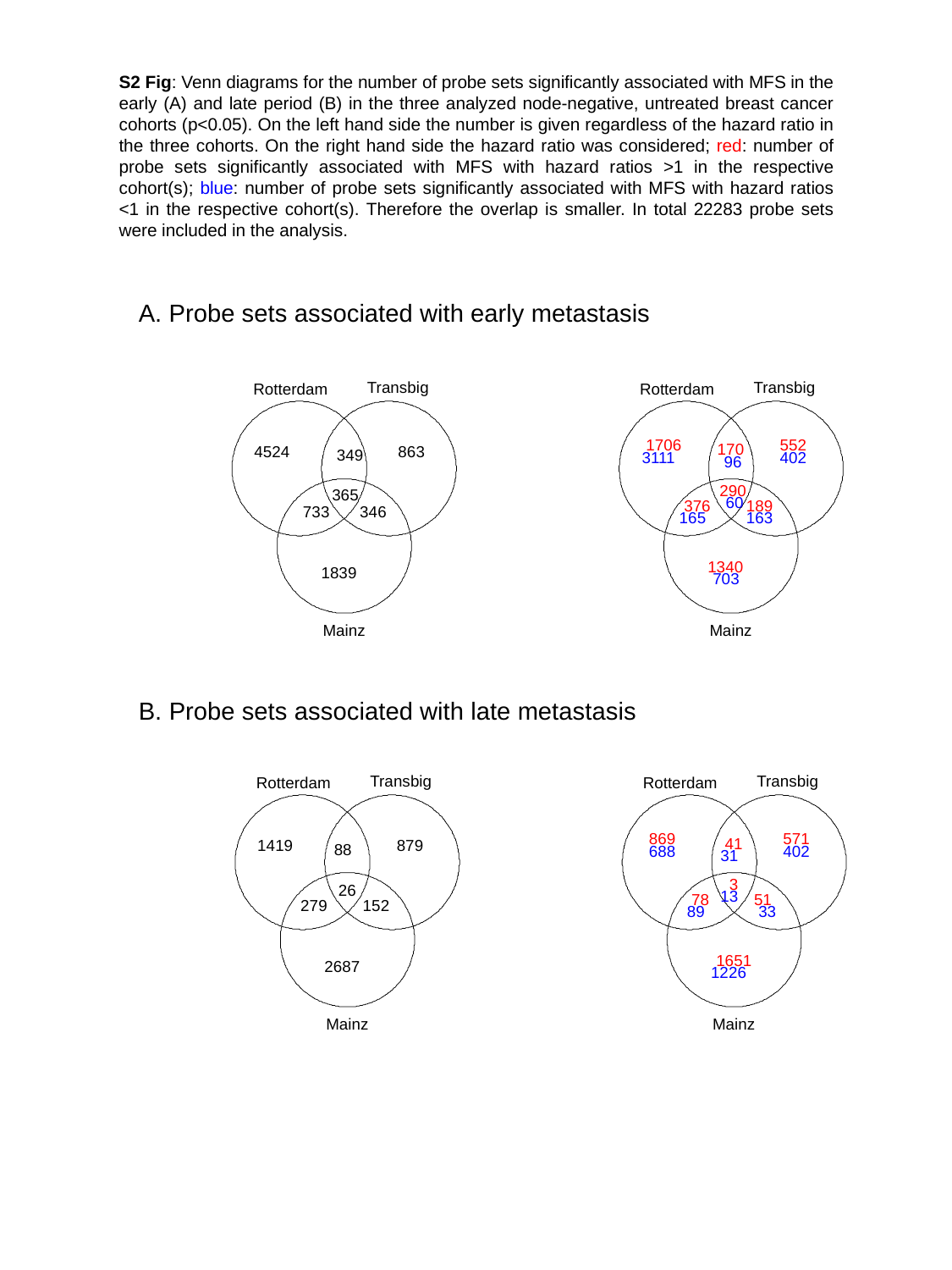

S2 Fig: Venn diagrams for the number of probe sets significantly associated with MFS in the early (A) and late period (B) in the three analyzed node-negative, untreated breast cancer cohorts (p<0.05). On the left hand side the number is given regardless of the hazard ratio in the three cohorts. On the right hand side the hazard ratio was considered; red: number of probe sets significantly associated with MFS with hazard ratios >1 in the respective cohort(s); blue: number of probe sets significantly associated with MFS with hazard ratios <1 in the respective cohort(s). Therefore the overlap is smaller. In total 22283 probe sets were included in the analysis.
A. Probe sets associated with early metastasis
Transbig
Transbig
Rotterdam
Rotterdam
1706
552
170
4524
863
349
3111
402
96
290
365
60
376
189
733
346
165
163
1340
1839
703
Mainz
Mainz
B. Probe sets associated with late metastasis
Transbig
Transbig
Rotterdam
Rotterdam
869
571
41
1419
879
88
688
402
31
3
26
13
78
51
279
152
89
33
1651
2687
1226
Mainz
Mainz
