# Supplementary material for: Epsin Family Member 3 and Ribosome-Related Genes Are Associated with Late Metastasis in Estrogen Receptor-Positive Breast Cancer and Long-Term Survival in Non-Small Cell Lung Cancer Using a Genome-Wide Identification and Validation Strategy
Source: PLoS One. 2016 Dec 7;11(12):e0167585. doi: 10.1371/journal.pone.0167585 (PMC5142791; doi:10.1371/journal.pone.0167585)

**A: RPS6 (200081_s_at)**

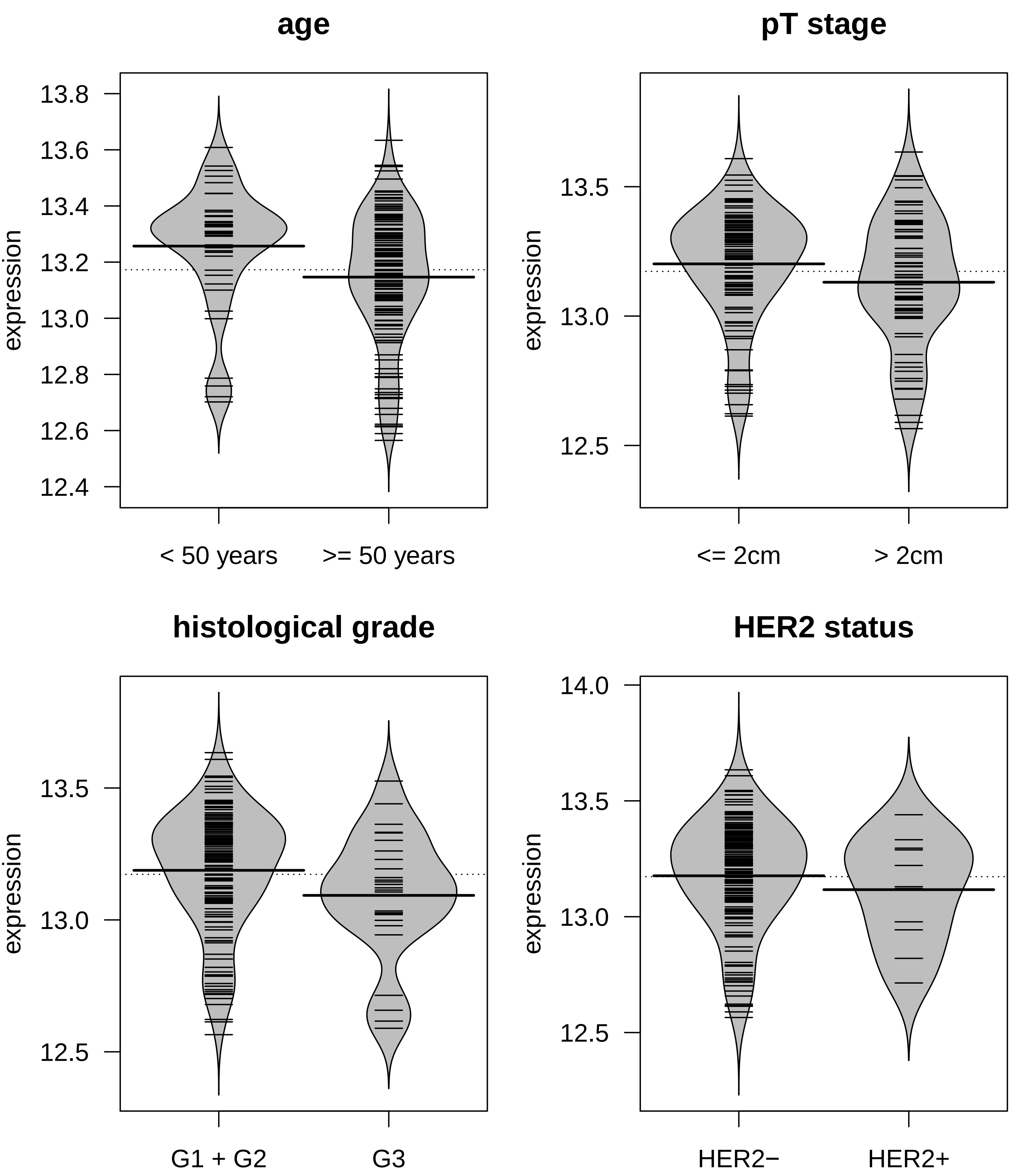

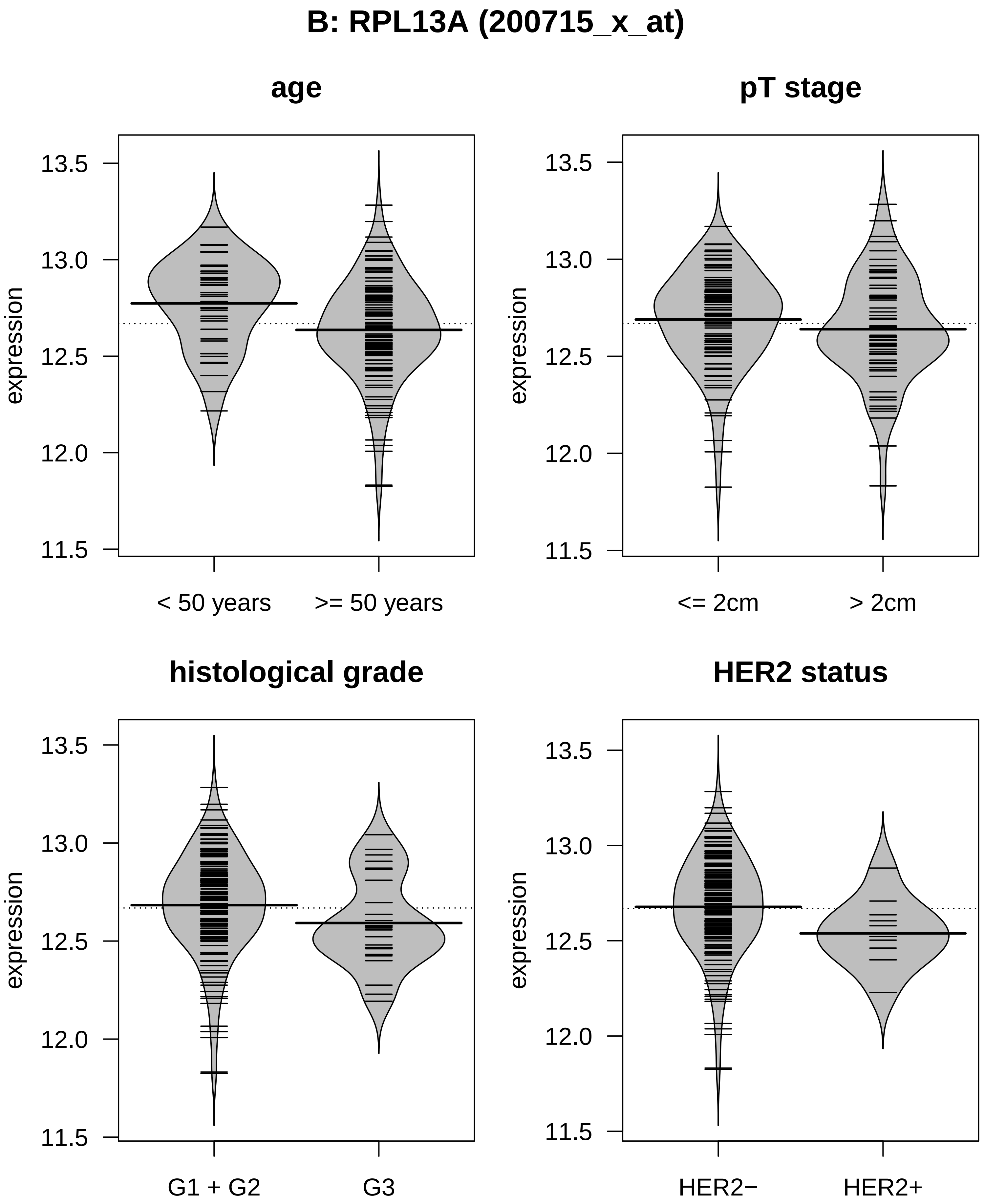
B: RPL13A (200715_x_at)
age
pT stage
histological grade
HER2 status
expression
13.5
13.0
12.5
12.0
11.5
< 50 years
>= 50 years
<= 2cm
> 2cm
G1 + G2
G3
HER2−
HER2+

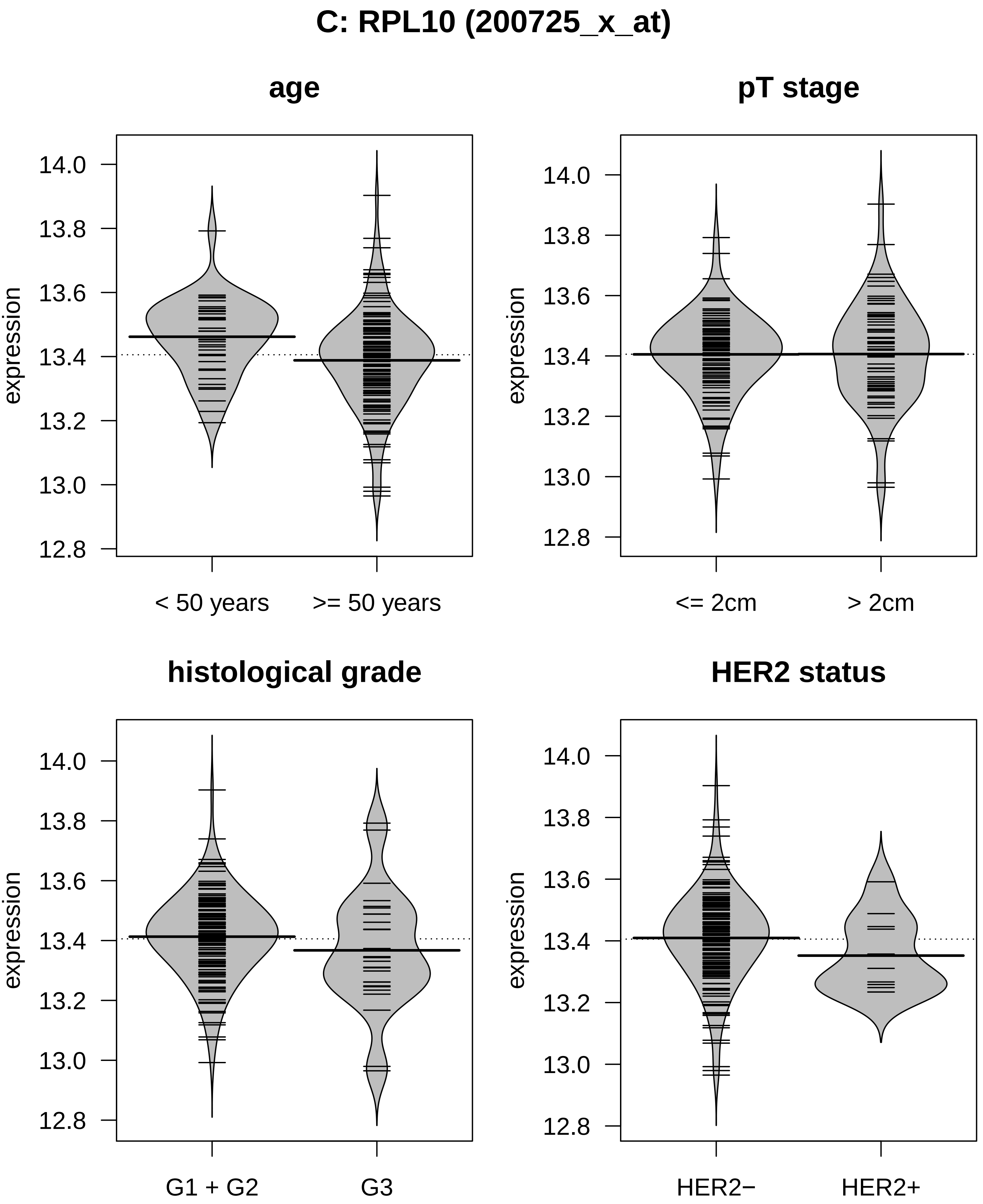
C: RPL10 (200725_x_at)
age
expression
< 50 years
>= 50 years
pT stage
<= 2cm
> 2cm
histological grade
G1 + G2
G3
HER2 status
HER2−
HER2+
14.0
13.8
13.6
13.4
13.2
13.0
12.8

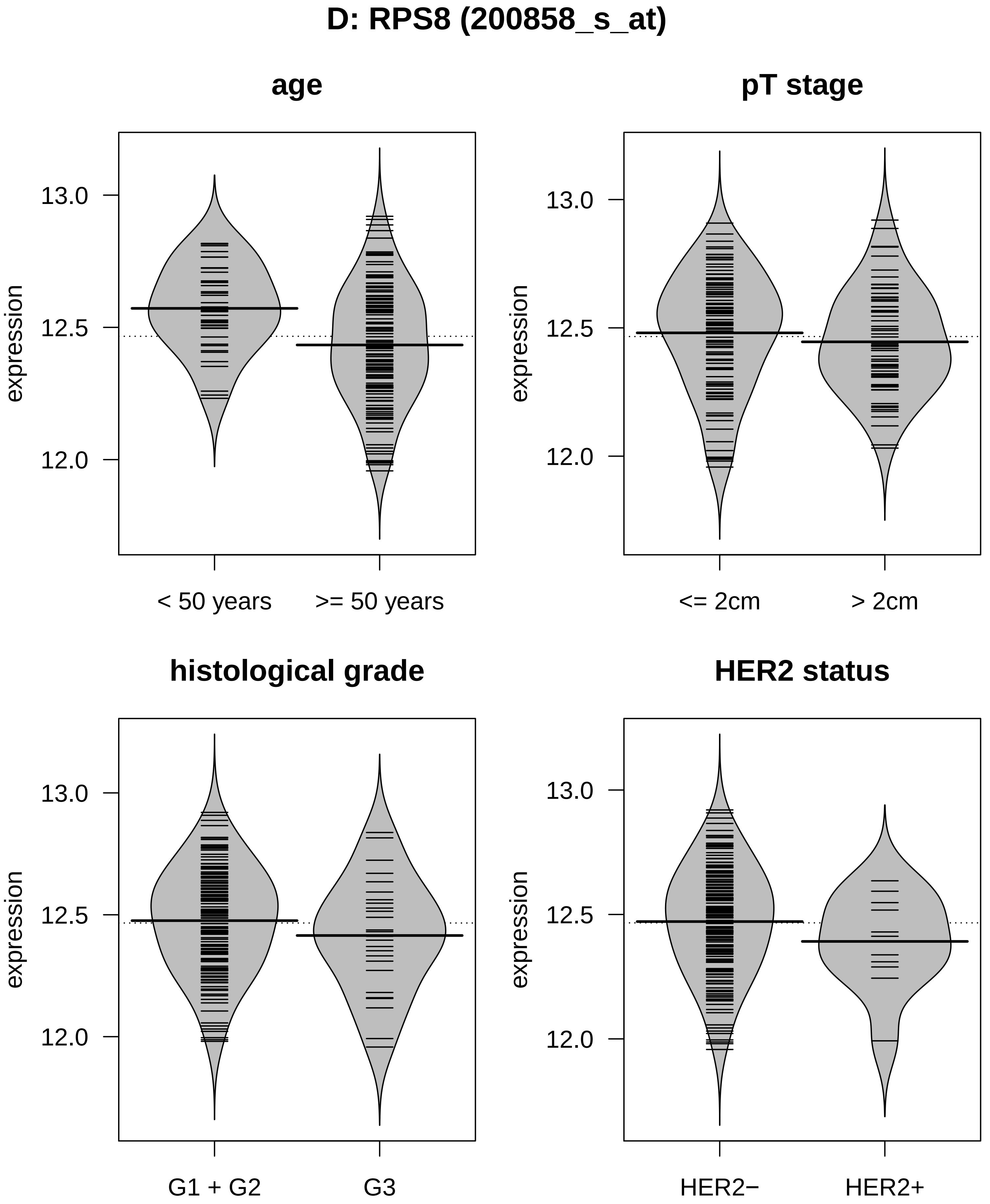

D: RPS8 (200858_s_at)
age
pT stage
histological grade
HER2 status
expression
13.0
12.5
12.0
< 50 years
>= 50 years
<= 2cm
> 2cm
G1 + G2
G3
HER2−
HER2+

E: RPL5 (200937_s_at)

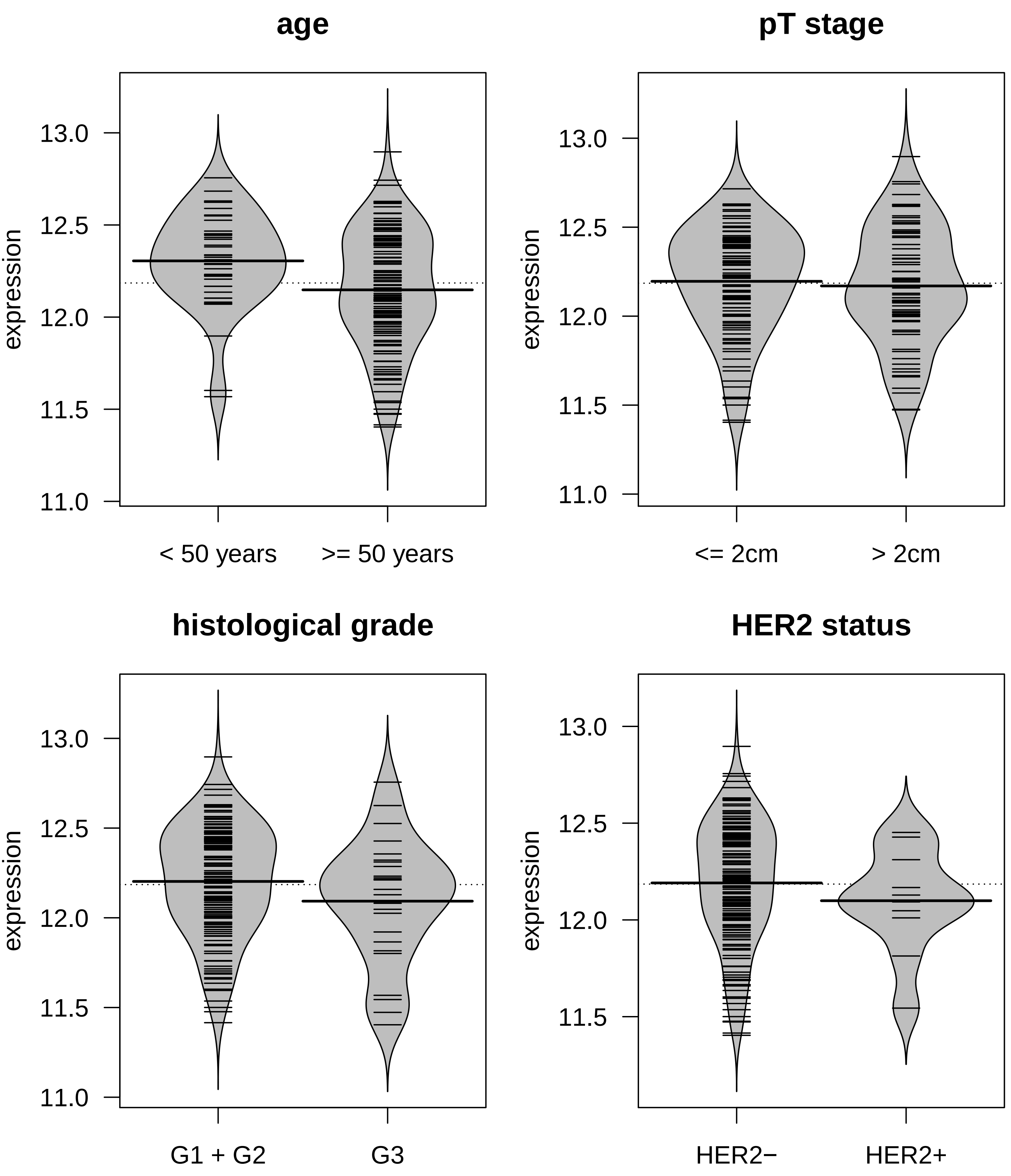

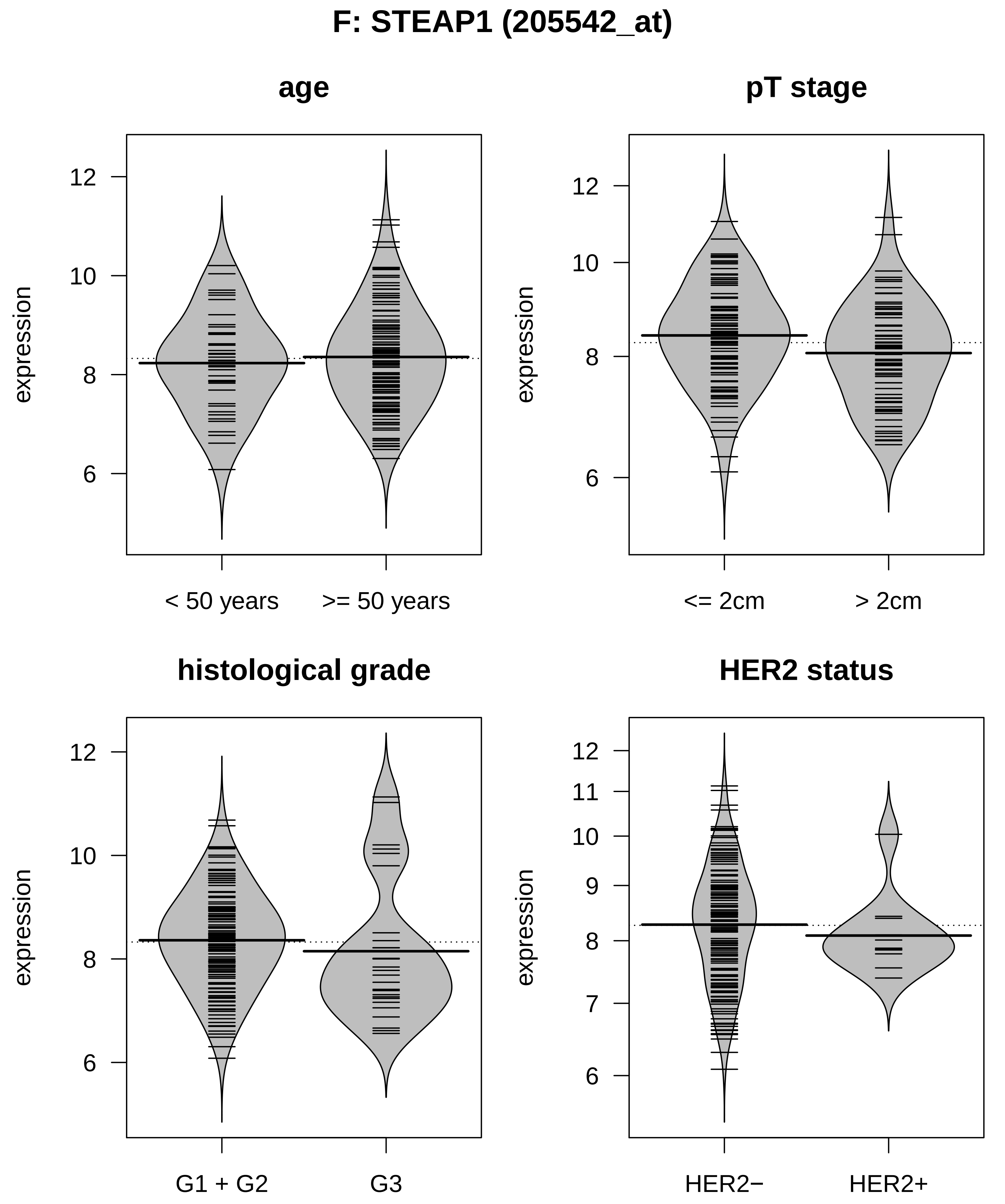
F: STEAP1 (205542_at)
age
pT stage
histological grade
HER2 status
expression
< 50 years
>= 50 years
<= 2cm
> 2cm
G1 + G2
G3
HER2−
HER2+

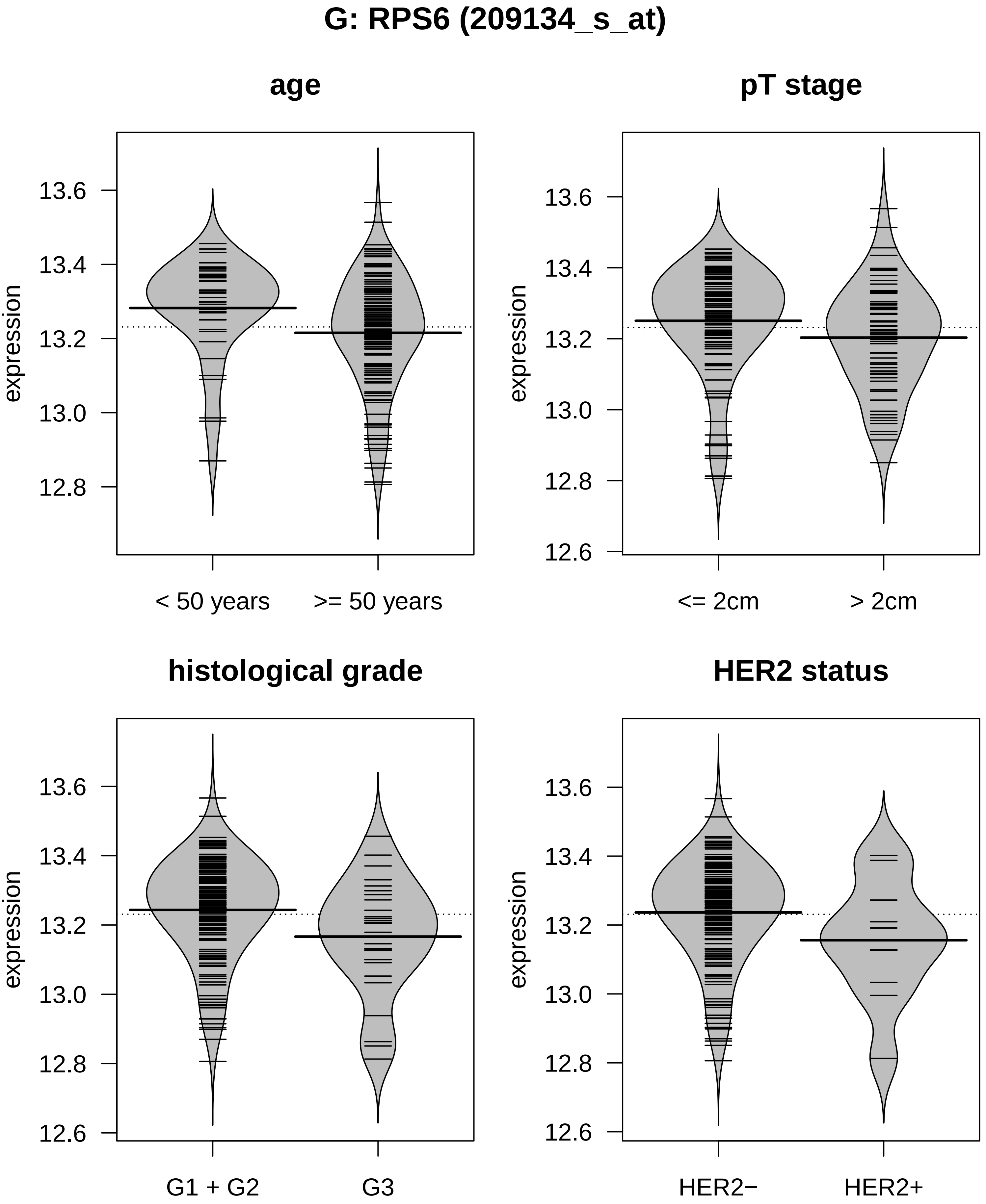
G: RPS6 (209134_s_at)
age
pT stage
histological grade
HER2 status
expression
13.6
13.4
13.2
13.0
12.8
12.6
< 50 years
>= 50 years
<= 2cm
> 2cm
G1 + G2
G3
HER2−
HER2+

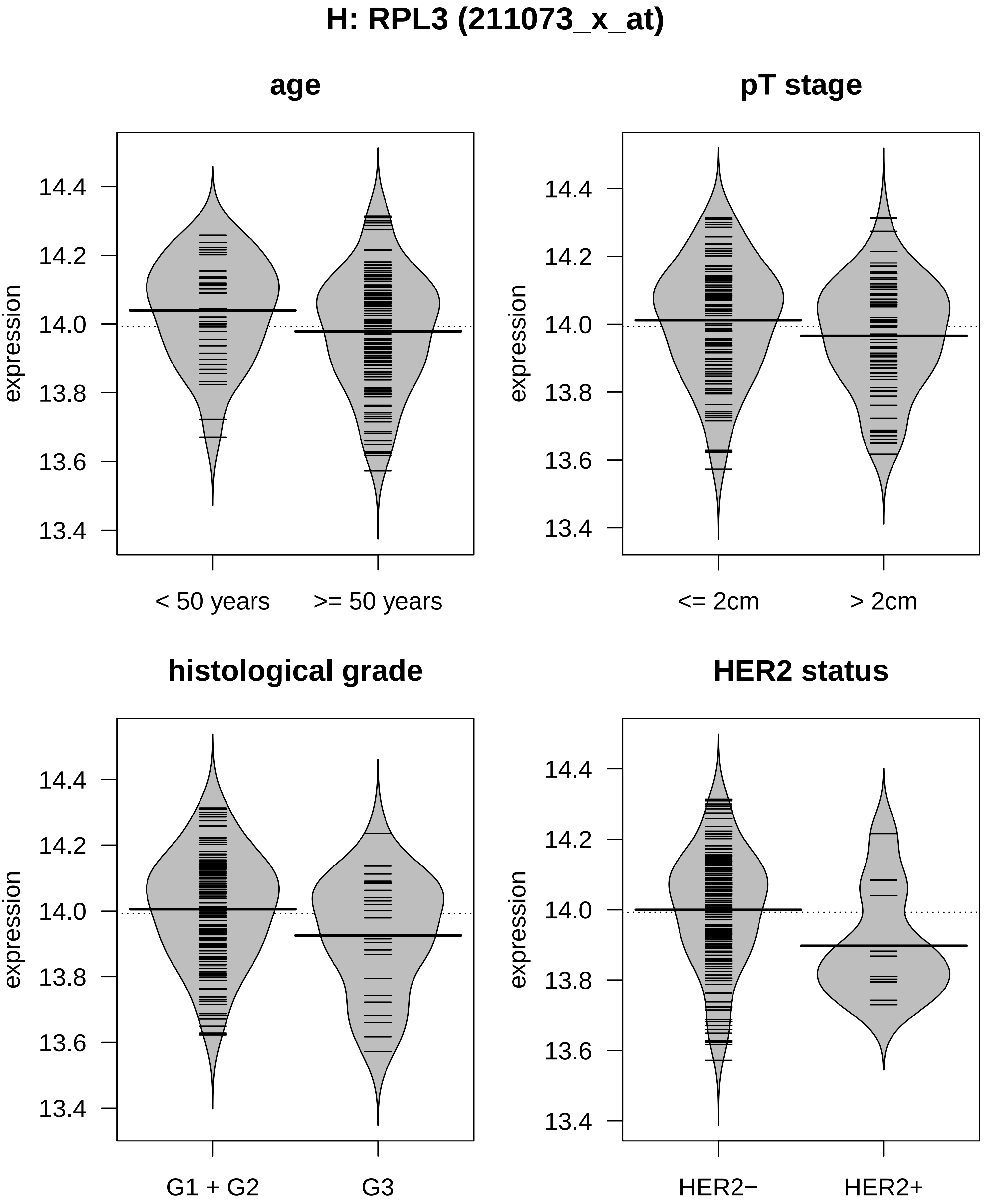
H: RPL3 (211073_x_at)
age
pT stage
histological grade
HER2 status
expression
14.4
14.2
14.0
13.8
13.6
13.4
< 50 years
>= 50 years
<= 2cm
> 2cm
G1 + G2
G3
HER2−
HER2+

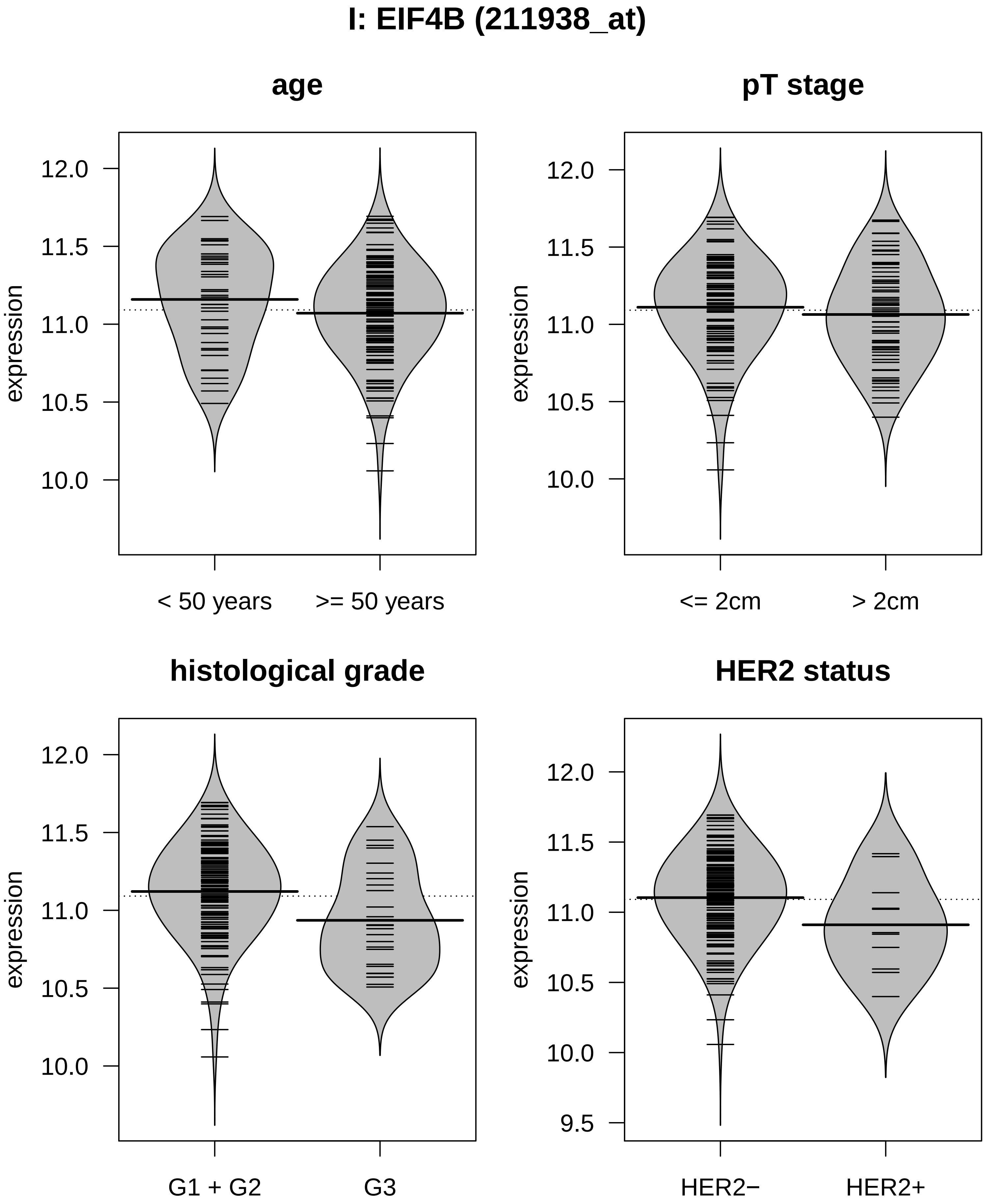
I: EIF4B (211938_at)
age
pT stage
histological grade
HER2 status
expression
12.0
11.5
11.0
10.5
10.0
9.5
< 50 years
>= 50 years
<= 2cm
> 2cm
G1 + G2
G3
HER2−
HER2+

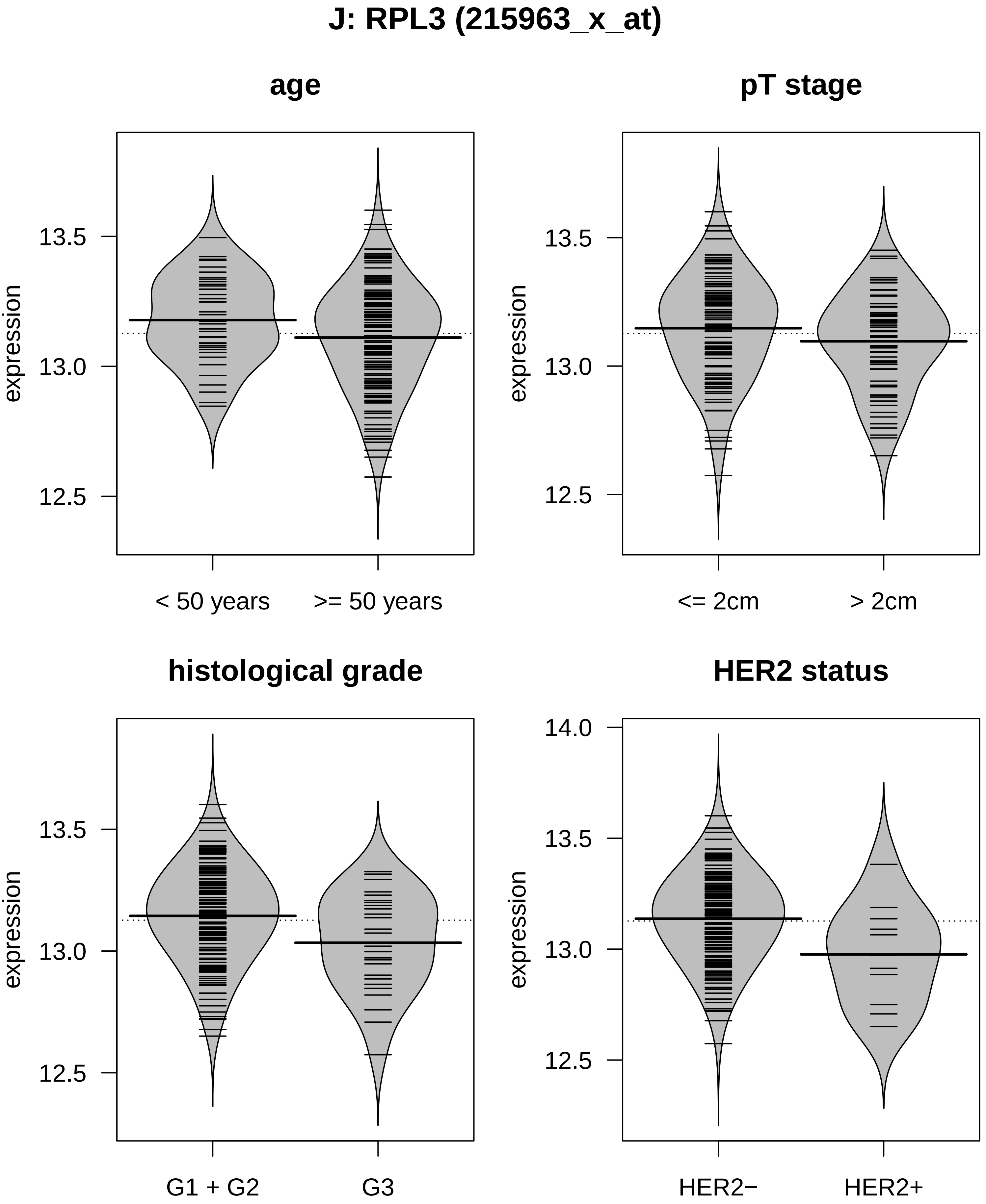

J: RPL3 (215963_x_at)
age
pT stage
histological grade
HER2 status
expression
14.0
13.5
13.0
12.5
< 50 years
>= 50 years
<= 2cm
> 2cm
G1 + G2
G3
HER2−
HER2+

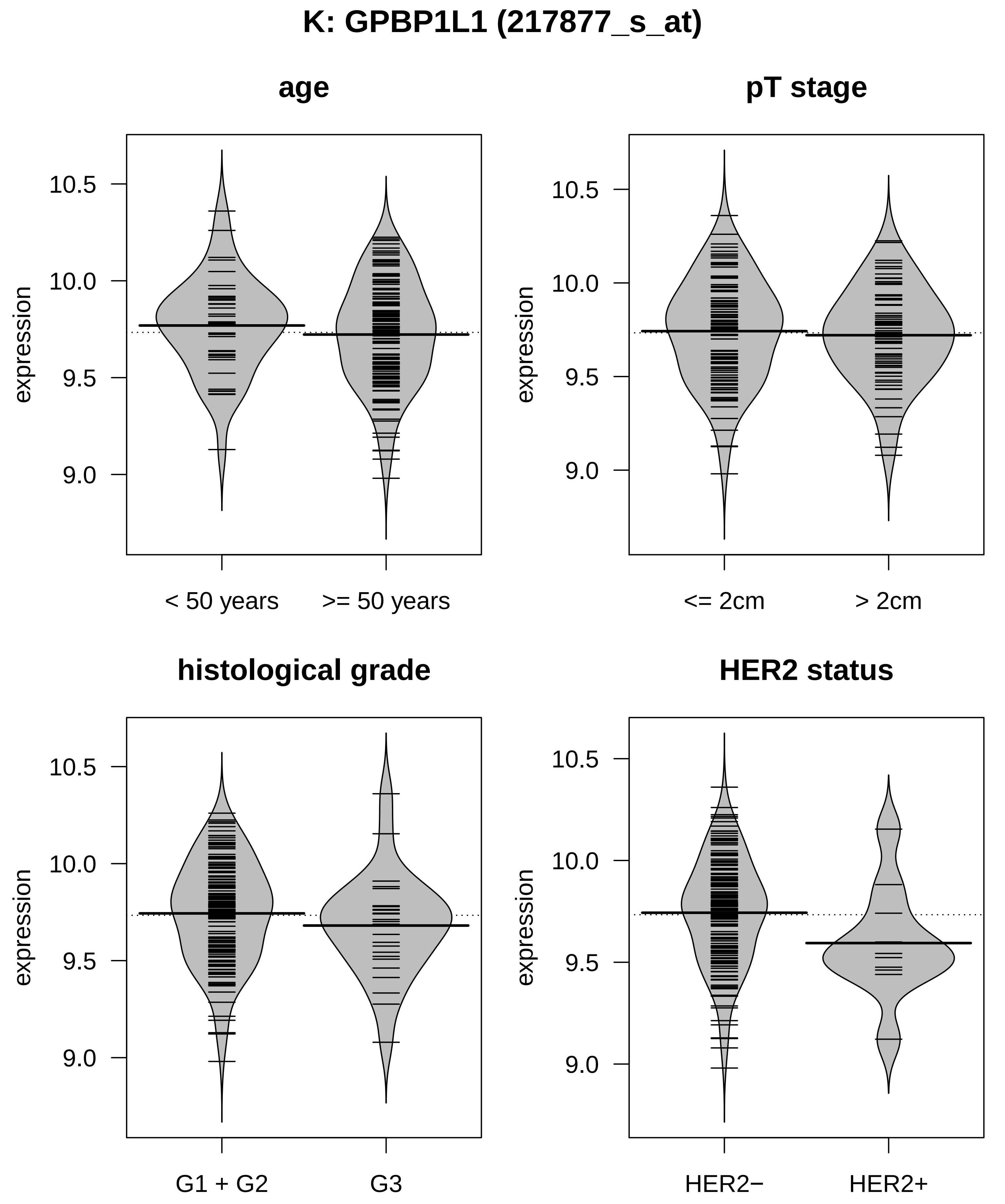

K: GPBP1L1 (217877_s_at)
age
expression
10.5
10.0
9.5
9.0
< 50 years
>= 50 years
pT stage
<= 2cm
> 2cm
histological grade
G1 + G2
G3
HER2 status
HER2−
HER2+

## L: EPN3 (220318_at)

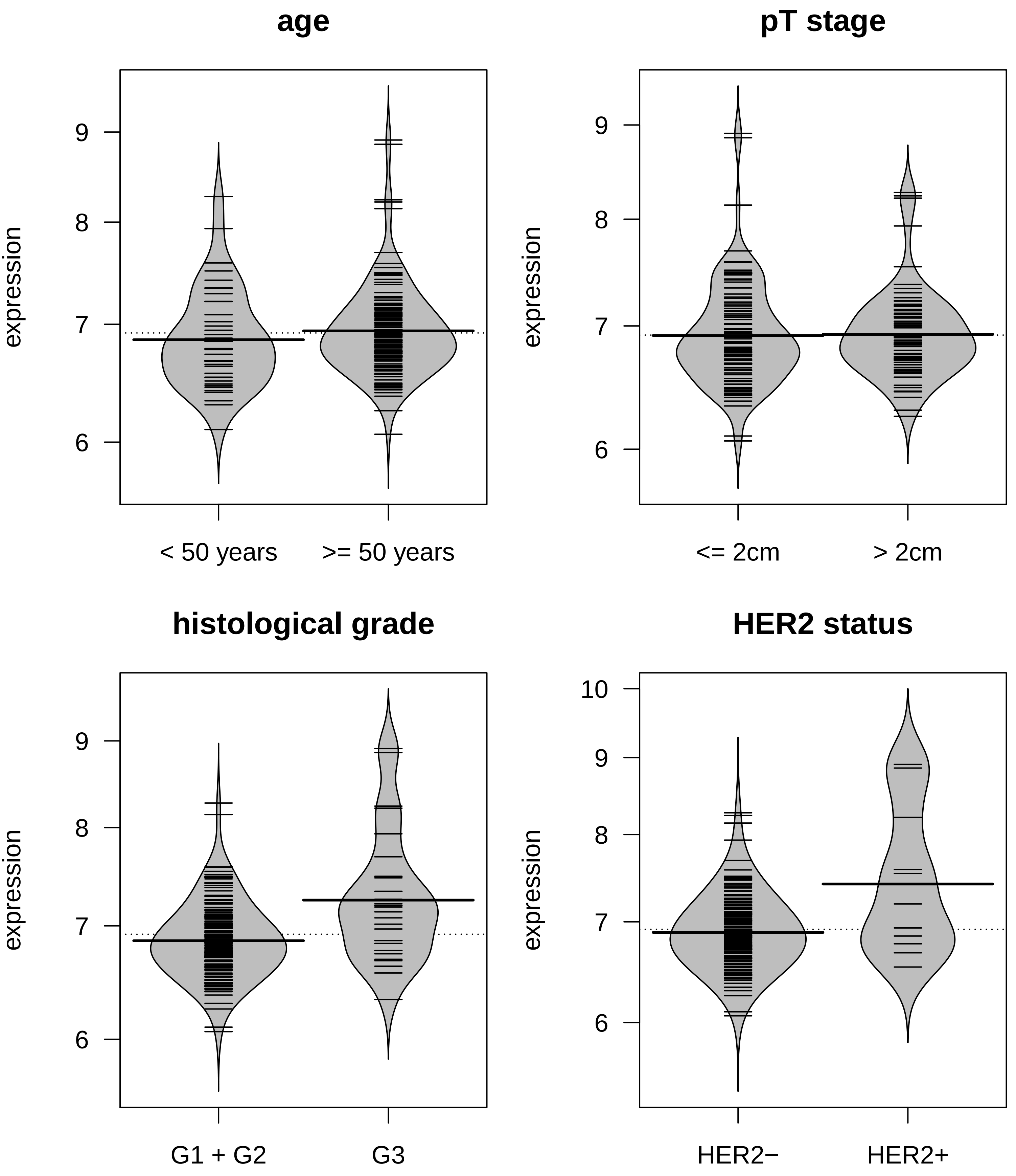

Supplement: S3 Fig — Beanplots of expression values of the validated late-type genes in association with age, stage, histological grade and HER2 status. (PDF) [file pone.0167585.s003.pdf]
