# Supplementary material for: Epsin Family Member 3 and Ribosome-Related Genes Are Associated with Late Metastasis in Estrogen Receptor-Positive Breast Cancer and Long-Term Survival in Non-Small Cell Lung Cancer Using a Genome-Wide Identification and Validation Strategy
Source: PLoS One. 2016 Dec 7;11(12):e0167585. doi: 10.1371/journal.pone.0167585 (PMC5142791; doi:10.1371/journal.pone.0167585)
Supplement: S1 Supporting Information — (DOCX) [file pone.0167585.s004.docx]

**S1 Supporting Information:** Summary over all analyzed datasets (ER-positive, tamoxifen-treated breast cancer, non-small cell lung cancer, ovarian cancer and colon cancer) and the data curation process.

**Curation of publicly available ER+ tamoxifen breast cancer datasets**

All breast cancer datasets were checked for duplicates and a pair of patients was considered duplicates when the correlation of their expression values was ≥ 0.999. Duplicates were removed and only ER+ patients who received adjuvant tamoxifen therapy and had available MFS data were included in the analysis.


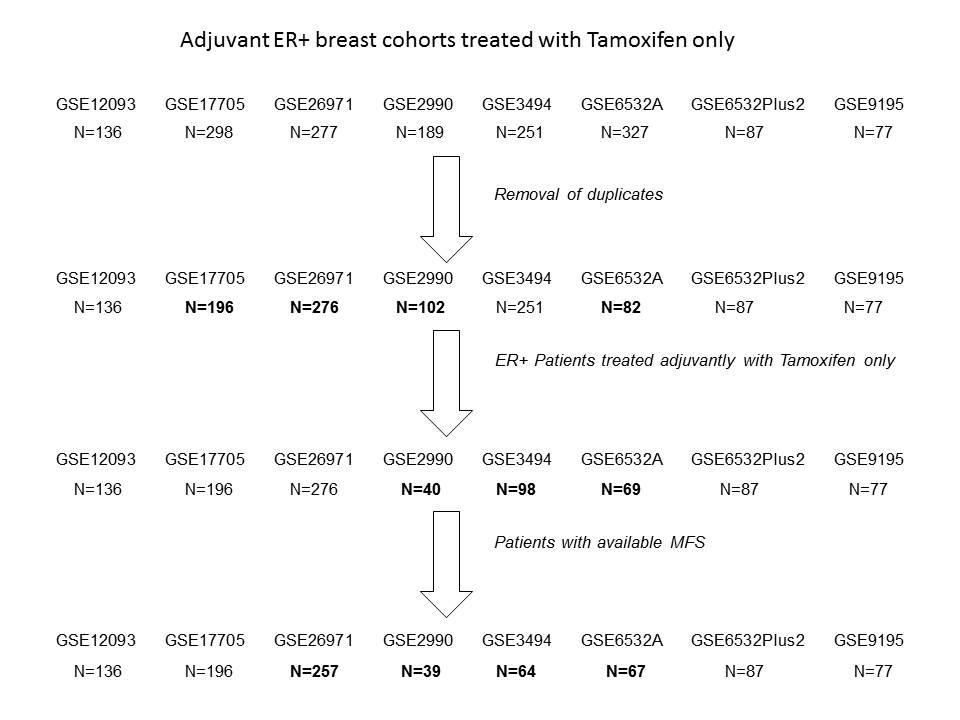


REFERENCES

- GSE12093

Zhang Y, Sieuwerts AM, McGreevy M, Casey G, Cufer T, Paradiso A, et al. The 76-gene signature defines high-risk patients that benefit from adjuvant tamoxifen therapy. Breast Cancer Res Treat. 2009;116(2):303-9.

- GSE17705

Symmans WF, Hatzis C, Sotiriou C, Andre F, Peintinger F, Regitnig P, et al. Genomic index of sensitivity to endocrine therapy for breast cancer. J Clin Oncol. 2010;28(27):4111-9.

- GSE26971

Filipits M, Rudas M, Jakesz R, Dubsky P, Fitzal F, Singer CF, et al. A new molecular predictor of distant recurrence in ER-positive, HER2-negative breast cancer adds independent information to conventional clinical risk factors. Clin Cancer Res. 2011;17(18):6012-20.

- GSE2990

Sotiriou C, Wirapati P, Loi S, Harris A, Fox S, Smeds J, et al. Gene expression profiling in breast cancer: understanding the molecular basis of histologic grade to improve prognosis. J Natl Cancer Inst. 2006;98(4):262-72.

- GSE3494

Miller LD, Smeds J, George J, Vega VB, Vergara L, Ploner A, et al. An expression signature for p53 status in human breast cancer predicts mutation status, transcriptional effects, and patient survival. Proc Natl Acad Sci U S A. 2005;102(38):13550-5.

- GSE6532

Loi S, Haibe-Kains B, Desmedt C, Lallemand F, Tutt AM, Gillet C, et al. Definition of clinically distinct molecular subtypes in estrogen receptor-positive breast carcinomas through genomic grade. J Clin Oncol. 2007;25:1239–46.

Loi S, Haibe-Kains B, Desmedt C, Wirapati P, Lallemand F, Tutt AM, et al. Predicting prognosis using molecular profiling in estrogen receptor-positive breast cancer treated with tamoxifen. BMC Genomics. 2008;9:239.

Loi S, Haibe-Kains B, Majjaj S, Lallemand F, Durbecq V, Larsimont D, et al. PIK3CA mutations associated with gene signature of low mTORC1 signaling and better outcomes in estrogen receptor-positive breast cancer. Proc Natl Acad Sci U S A. 2010;107(22):10208-13.

- GSE9195

Loi S, Haibe-Kains B, Desmedt C, Wirapati P, Lallemand F, Tutt AM, et al. Predicting prognosis using molecular profiling in estrogen receptor-positive breast cancer treated with tamoxifen. BMC Genomics. 2008;9:239.

Loi S, Haibe-Kains B, Majjaj S, Lallemand F, Durbecq V, Larsimont D, et al. PIK3CA mutations associated with gene signature of low mTORC1 signaling and better outcomes in estrogen receptor-positive breast cancer. Proc Natl Acad Sci U S A. 2010;107(22):10208-13.

**Curation of publicly available NSCLC datasets**

Normal (non-tumoral) samples and small cell carcinomas were removed: 65 normal samples in GSE19188, 20 normal samples in GSE31210, 14 normal samples and 24 small cell carcinomas in GSE30219. For dataset Shedden et al. (2008) 444 overlapping patients with clinical and expression data were used. All datasets were checked for duplicates as described above for the ER+ tamoxifen breast cancer datasets. Shedden had 43 duplicates with GSE14814 which were removed in the latter. In GSE37745 two samples belonging to the same patient but with different clinical data were both removed. Only patients with available OS data were included in the analysis.

***
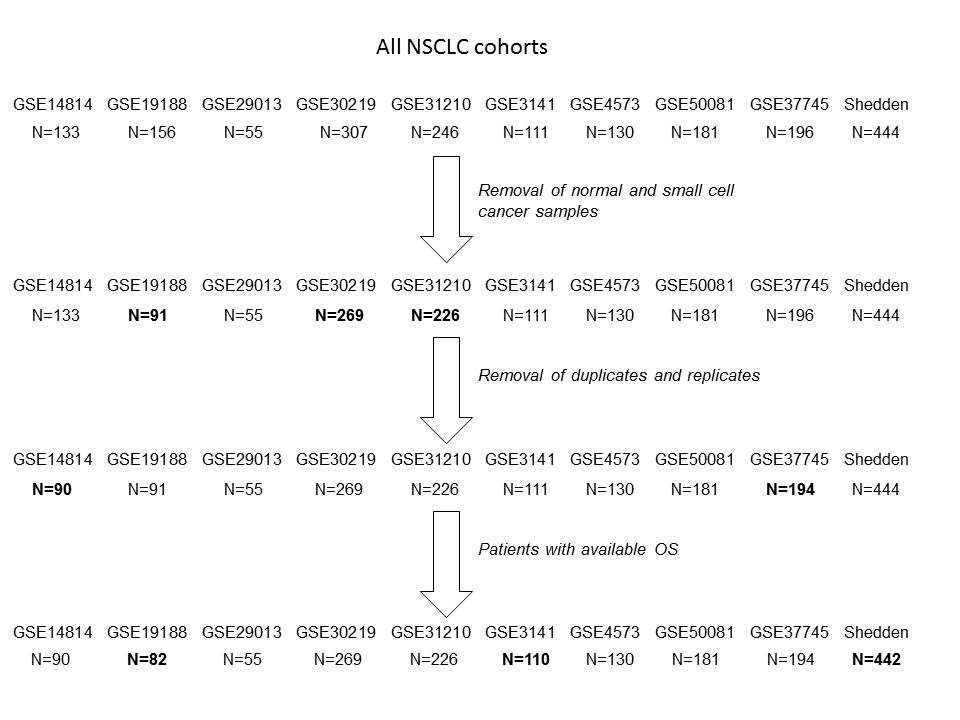
***

REFERENCES

- GSE14814

Zhu CQ, Ding K, Strumpf D, Weir BA, Meyerson M, Pennell N, et al. Prognostic and predictive gene signature for adjuvant chemotherapy in resected non-small-cell lung cancer. J Clin Oncol. 2010;28(29):4417-24.

- GSE19188

Hou J, Aerts J, den Hamer B, van Ijcken W, den Bakker M, Riegman P, et al. Gene expression-based classification of non-small cell lung carcinomas and survival prediction. PLoS One. 2010;5(4):e10312.

- GSE29013

Xie Y, Xiao G, Coombes KR, Behrens C, Solis LM, Raso G, et al. Robust gene expression signature from formalin-fixed paraffin-embedded samples predicts prognosis of non-small-cell lung cancer patients. Clin Cancer Res. 2011;17(17):5705-14.

- GSE30219

Rousseaux S, Debernardi A, Jacquiau B, Vitte AL, LeBescont A, Vesin A, et al. Ectopic activation of germline and placental genes identifies aggressive metastasis-prone lung cancers. Sci Transl Med. 2013;5(186):186ra66.

- GSE31210

Okayama H, Kohno T, Ishii Y, Shimada Y, Shiraishi K, Iwakawa R, et al. Identification of genes upregulated in ALK-positive and EGFR/KRAS/ALK-negative lung adenocarcinomas. Cancer Res. 2012;72(1):100-11.

Yamauchi M, Yamaguchi R, Nakata A, Kohno T, Nagasaki M, Shimamura T, et al. Epidermal growth factor receptor tyrosine kinase defines critical prognostic genes of stage I lung adenocarcinoma. PLoS One. 2012;7(9):e43923.

- GSE3141

Bild AH, Yao G, Chang JT, Wang Q, Potti A, Chasse D, et al. Oncogenic pathway signatures in human cancers as a guide to targeted therapies. Nature. 2006;439(7074):353-7.

- GSE4573

Raponi M, Zhang Y, Yu J, Chen G, Lee G, Taylor JM, et al. Gene expression signatures for predicting prognosis of squamous cell and adenocarcinomas of the lung. Cancer Res. 2006;66(15):7466-72.

- GSE50081

Der SD, Sykes J, Pintilie M, Zhu CQ, Strumpf D, Liu N, et al. Validation of a histology-independent prognostic gene signature for early-stage, non-small-cell lung cancer including stage IA patients. J Thorac Oncol. 2014;9(1):59-64.

- GSE37745

Botling J, Edlund K, Lohr M, Hellwig B, Holmberg L, Lambe M, et al. Biomarker discovery in non-small cell lung cancer: integrating gene expression profiling, meta-analysis, and tissue microarray validation. Clin Cancer Res. 2013;19(1):194-204.

- Shedden

Shedden K, Taylor JM, Enkemann SA, Tsao MS, Yeatman TJ, Gerald WL, et al. Gene expression-based survival prediction in lung adenocarcinoma: a multi-site, blinded validation study. Nat Med. 2008;14:822-7.

**Curation of publicly available ovarian cancer datasets**

Normal (non-tumoral) samples were removed and patients from all cohorts were checked for duplicates as described above. Only samples with gene expression data on the Affymetrix HG U133A and Plus2 array and with available OS data were included in the analysis.


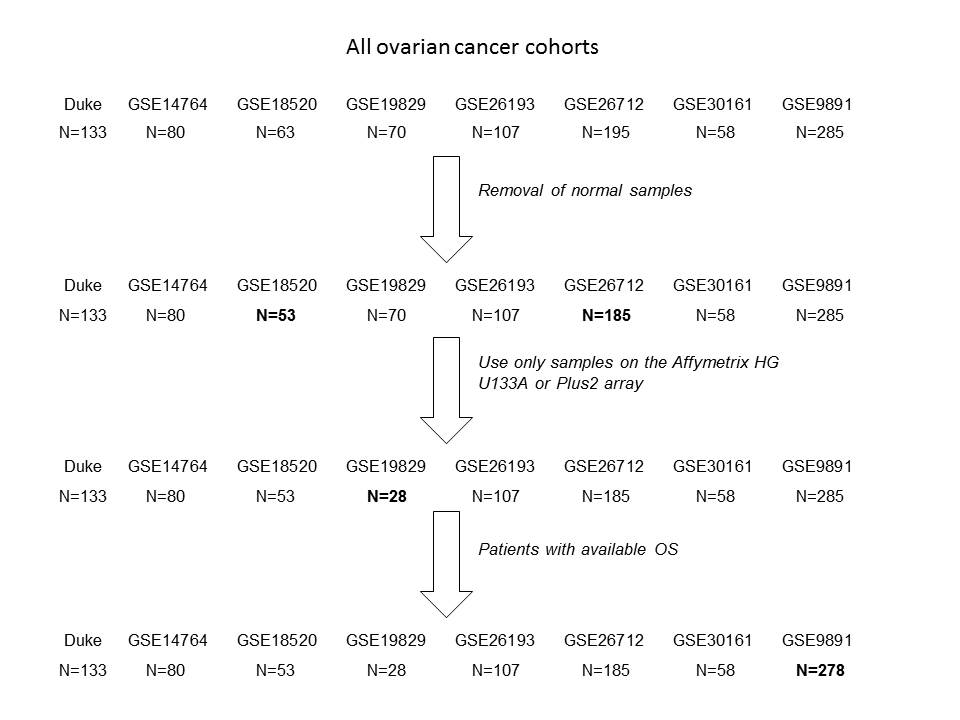


REFERENCES

- Duke

Dressman HK, Berchuck A, Chan G, Zhai J, Bild A, Sayer R, et al. An integrated genomic-based approach to individualized treatment of patients with advanced-stage ovarian cancer. J Clin Oncol. 2007; 25(5):517-25.

- GSE14764

Denkert C, Budczies J, Darb-Esfahani S, Györffy B, Sehouli J, Könsgen D, et al. A prognostic gene expression index in ovarian cancer - validation across different independent data sets. J Pathol. 2009;218(2):273-80.

- GSE18520

Mok SC, Bonome T, Vathipadiekal V, Bell A, Johnson ME, Wong KK, et al. A gene signature predictive for outcome in advanced ovarian cancer identifies a survival factor: microfibril-associated glycoprotein 2. Cancer Cell. 2009;16(6):521-32.

- GSE19829

Konstantinopoulos PA, Spentzos D, Karlan BY, Taniguchi T, Fountzilas E, Francoeur N, et al. Gene expression profile of BRCAness that correlates with responsiveness to chemotherapy and with outcome in patients with epithelial ovarian cancer. J Clin Oncol. 2010;28(22):3555-61.

- GSE26193

Mateescu B, Batista L, Cardon M, Gruosso T, de Feraudy Y, Mariani O, et al. miR-141 and miR-200a act on ovarian tumorigenesis by controlling oxidative stress response. Nat Med. 2011;17(12):1627-35.

- GSE26712

Bonome T, Levine DA, Shih J, Randonovich M, Pise-Masison CA, Bogomolniy F, et al. A gene signature predicting for survival in suboptimally debulked patients with ovarian cancer. Cancer Res. 2008;68(13):5478-86.

Vathipadiekal V, Wang V, Wei W, Waldron L, Drapkin R, Gillette M, et al. Creation of a Human Secretome: A Novel Composite Library of Human Secreted Proteins: Validation Using Ovarian Cancer Gene Expression Data and a Virtual Secretome Array. Clin Cancer Res. 2015;21(21):4960-9.

- GSE30161

Ferriss JS, Kim Y, Duska L, Birrer M, Levine DA, Moskaluk C, et al. Multi-gene expression predictors of single drug responses to adjuvant chemotherapy in ovarian carcinoma: predicting platinum resistance. PLoS One. 2012;7(2):e30550.

- GSE9891

Tothill RW, Tinker AV, George J, Brown R, Fox SB, Lade S, et al. Novel molecular subtypes of serous and endometrioid ovarian cancer linked to clinical outcome. Clin Cancer Res. 2008;14(16):5198-208.

**Curation of publicly available colon cancer datasets**

All cohorts were checked for duplicates and only patients with available DFS data were used for the analysis.


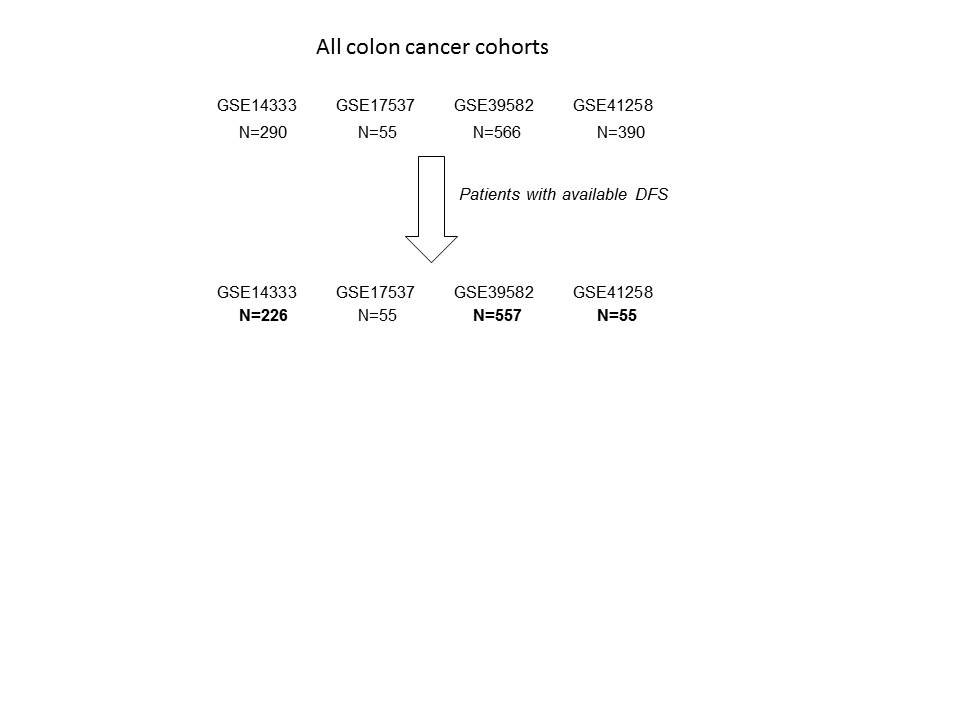


REFERENCES

- GSE14333

Jorissen RN, Gibbs P, Christie M, Prakash S, Lipton L, Desai J, et al. Metastasis-Associated Gene Expression Changes Predict Poor Outcomes in Patients with Dukes Stage B and C Colorectal Cancer. Clin Cancer Res. 2009;15(24):7642-7651.

- GSE17537

Smith JJ, Deane NG, Wu F, Merchant NB, Zhang B, Jiang A, et al. Experimentally derived metastasis gene expression profile predicts recurrence and death in patients with colon cancer. Gastroenterology. 2010;138(3):958-68.

Freeman TJ, Smith JJ, Chen X, Washington MK, Roland JT, Means AL, et al. Smad4-mediated signaling inhibits intestinal neoplasia by inhibiting expression of β-catenin. Gastroenterology. 2012;142(3):562-571.e2.

- GSE39582

Marisa L, de Reyniès A, Duval A, Selves J, Gaub MP, Vescovo L, et al. Gene expression classification of colon cancer into molecular subtypes: characterization, validation, and prognostic value. PLoS Med. 2013;10(5):e1001453.

- GSE41258

Sheffer M, Bacolod MD, Zuk O, Giardina SF, Pincas H, Barany F, et al. Association of survival and disease progression with chromosomal instability: a genomic exploration of colorectal cancer. Proc Natl Acad Sci U S A. 2009;106(17):7131-6.
