# Supplementary material for: Epsin Family Member 3 and Ribosome-Related Genes Are Associated with Late Metastasis in Estrogen Receptor-Positive Breast Cancer and Long-Term Survival in Non-Small Cell Lung Cancer Using a Genome-Wide Identification and Validation Strategy
Source: PLoS One. 2016 Dec 7;11(12):e0167585. doi: 10.1371/journal.pone.0167585 (PMC5142791; doi:10.1371/journal.pone.0167585)
Supplement: S1 Table — Clinicopathological characteristics for all patients and for the subset of ER-positive patients included in the late analysis for the Mainz (A-B), Rotterdam (C-D), and Transbig (E-F) cohort. (DOCX) [file pone.0167585.s005.docx]

**S1 Table A:** Clinicopathological characteristics of node-negative breast cancer patients (fresh frozen tissue) from the Mainz cohort (n=200). Estrogen receptor, progesterone receptor and HER2 status were derived from RMA pre-processed gene array data. Cut-points were 8.2 for the estrogen receptor, 11.2 for HER2 and 4.5 for the progesterone receptor.

|  | **All patients** | | **ER positive** | |
| --- | --- | --- | --- | --- |
| Characteristics | **n** | **%** | **n** | **%** |
| **Age at diagnosis** |  |  |  |  |
| <50 | 49 | 24.5 | 40 | 23.7 |
| ≥50 | 151 | 75.5 | 129 | 76.3 |
| **pT stage** |  |  |  |  |
| ≤2cm | 112 | 56.0 | 100 | 59.2 |
| >2cm | 88 | 44.0 | 69 | 40.8 |
| **Histological grade** |  |  |  |  |
| G I | 42 | 21.0 | 42 | 24.9 |
| G II | 109 | 54.5 | 100 | 59.2 |
| G III | 49 | 24.5 | 27 | 15.4 |
| **Estrogen receptor status** |  |  |  |  |
| RNA expression low | 31 | 15.5 | - | - |
| RNA expression high | 169 | 84.5 | - | - |
| **Progesterone receptor status** |  |  |  |  |
| RNA expression low | 86 | 43.0 | 55 | 32.5 |
| RNA expression high | 114 | 57.0 | 114 | 67.5 |
| **Hormone receptor status**^1^ |  |  |  |  |
| RNA expression low | 31 | 15.5 | - | - |
| RNA expression high | 169 | 84.5 | - | - |
| **HER2 status** |  |  |  |  |
| RNA expression low | 181 | 90.5 | 158 | 93.5 |
| RNA expression high | 19 | 9.5 | 11 | 6.5 |
| **Survival** |  |  |  |  |
| Dead | 57 | 28.5 | 57 | 28.5 |
| Alive | 143 | 71.5 | 143 | 71.5 |
| **Metastasis** |  |  |  |  |
| Yes | 47 | 23.5 | 36 | 21.3 |
| No | 153 | 76.5 | 133 | 78.7 |
| **Relapse** |  |  |  |  |
| Yes | 58 | 29.0 | 45 | 26.6 |
| No | 142 | 71.0 | 124 | 73.4 |

^1^The hormone receptor status is defined as positive when one of either the estrogen or the progesterone receptor status, is positive.

**S1 Table B:** Clinicopathological characteristics of node-negative breast cancer patients (fresh frozen tissue) from the Mainz cohort who did not develop a metastasis in the first three years after surgery (n=172).

|  | **All patients** | | **ER positive** | |
| --- | --- | --- | --- | --- |
| Characteristics | **n** | **%** | **n** | **%** |
| **Age at diagnosis** |  |  |  |  |
| <50 | 42 | 24.4 | 37 | 25.0 |
| ≥50 | 130 | 75.6 | 111 | 75.0 |
| **pT stage** |  |  |  |  |
| ≤2cm | 101 | 58.7 | 92 | 62.2 |
| >2cm | 71 | 41.3 | 56 | 37.8 |
| **Histological grade** |  |  |  |  |
| G I | 41 | 23.8 | 41 | 27.7 |
| G II | 96 | 55.8 | 88 | 59.5 |
| G III | 35 | 20.3 | 19 | 12.8 |
| **Estrogen receptor status** |  |  |  |  |
| RNA expression low | 24 | 14.0 | - | - |
| RNA expression high | 148 | 86.0 | - | - |
| **Progesterone receptor status** |  |  |  |  |
| RNA expression low | 69 | 40.1 | 45 | 30.4 |
| RNA expression high | 103 | 59.9 | 103 | 69.6 |
| **Hormone receptor status**^1^ |  |  |  |  |
| RNA expression low | 24 | 14.0 | - | - |
| RNA expression high | 148 | 86.0 | - | - |
| **HER2 status** |  |  |  |  |
| RNA expression low | 159 | 92.4 | 142 | 95.9 |
| RNA expression high | 13 | 7.6 | 6 | 4.1 |
| **Survival** |  |  |  |  |
| Dead | 36 | 20.9 | 36 | 20.9 |
| Alive | 136 | 79.1 | 136 | 79.1 |
| **Metastasis** |  |  |  |  |
| Yes | 28 | 16.3 | 23 | 15.5 |
| No | 144 | 83.7 | 125 | 84.5 |
| **Relapse** |  |  |  |  |
| Yes | 38 | 22.1 | 31 | 18.3 |
| No | 134 | 77.9 | 117 | 81.7 |

^1^The hormone receptor status is defined as positive when one of either the estrogen or the progesterone receptor status, is positive.

**S1 Table C:** Clinicopathological characteristics of node-negative breast cancer patients (fresh frozen tissue) from the Rotterdam cohort (n=286). Estrogen receptor, progesterone receptor and HER2 status were derived from RMA pre-processed gene array data. Cut-points were 8.2 for the estrogen receptor, 11.2 for HER2 and 4.5 for the progesterone receptor.

|  | **All patients** | | **ER positive** | |
| --- | --- | --- | --- | --- |
| Characteristics | **n** | **%** | **n** | **%** |
| **Estrogen receptor status** |  |  |  |  |
| RNA expression low | 78 | 27.3 | - | - |
| RNA expression high | 208 | 72.7 | - | - |
| **Progesterone receptor status** |  |  |  |  |
| RNA expression low | 158 | 55.2 | 82 | 39.4 |
| RNA expression high | 128 | 44.8 | 126 | 60.6 |
| **Hormone receptor status**^1^ |  |  |  |  |
| RNA expression low | 76 | 26.6 | - | - |
| RNA expression high | 210 | 73.4 | - | - |
| **HER2 status** |  |  |  |  |
| RNA expression low | 236 | 82.5 | 178 | 85.6 |
| RNA expression high | 50 | 17.5 | 30 | 14.4 |
| **Metastasis** |  |  |  |  |
| Yes | 107 | 37.4 | 79 | 38.0 |
| No | 179 | 62.6 | 129 | 62.0 |

^1^The hormone receptor status is defined as positive when one of either the estrogen or the progesterone receptor status, is positive.

**S1 Table D:** Clinicopathological characteristics of node-negative breast cancer patients (fresh frozen tissue) from the Rotterdam cohort who did not develop a metastasis in the first three years after surgery (n=217).

|  | **All patients** | | **ER positive** | |
| --- | --- | --- | --- | --- |
| Characteristics | **n** | **%** | **n** | **%** |
| **Estrogen receptor status** |  |  |  |  |
| RNA expression low | 52 | 24.0 | - | - |
| RNA expression high | 165 | 76.0 | - | - |
| **Progesterone receptor status** |  |  |  |  |
| RNA expression low | 109 | 50.2 | 96 | 58.2 |
| RNA expression high | 108 | 49.8 | 69 | 40.8 |
| **Hormone receptor status**^1^ |  |  |  |  |
| RNA expression low | 50 | 23.0 | - | - |
| RNA expression high | 167 | 77.0 | - | - |
| **HER2 status** |  |  |  |  |
| RNA expression low | 182 | 83.9 | 144 | 87.3 |
| RNA expression high | 35 | 16.1 | 21 | 12.7 |
| **Metastasis** |  |  |  |  |
| Yes | 38 | 17.5 | 36 | 21.8 |
| No | 179 | 82.5 | 129 | 78.2 |

^1^The hormone receptor status is defined as positive when one of either the estrogen or the progesterone receptor status, is positive.

**S1 Table E:** Clinicopathological characteristics of node-negative breast cancer patients (fresh frozen tissue) from the Transbig cohort (n=280). Estrogen receptor, progesterone receptor and HER2 status were derived from RMA pre-processed gene array data. Cut-points were 8.2 for the estrogen receptor, 11.2 for HER2 and 4.5 for the progesterone receptor.

|  | **All patients** | | **ER positive** | |
| --- | --- | --- | --- | --- |
| Characteristics | **n** | **%** | **n** | **%** |
| **Age at diagnosis** |  |  |  |  |
| <50 | 158 | 56.4 | 110 | 54.7 |
| ≥50 | 122 | 43.5 | 91 | 45.3 |
| **pT stage** |  |  |  |  |
| ≤2cm | 149 | 53.2 | 115 | 57.2 |
| >2cm | 131 | 46.8 | 86 | 42.8 |
| **Histological grade** |  |  |  |  |
| G I | 56 | 20.0 | 53 | 26.4 |
| G II | 109 | 38.9 | 91 | 45.3 |
| G III | 100 | 35.7 | 44 | 21.9 |
| Not documented | 15 | 5.4 | 13 | 6.5 |
| **Estrogen receptor status** |  |  |  |  |
| RNA expression low | 79 | 28.2 | - | - |
| RNA expression high | 201 | 71.8 | - | - |
| **Progesterone receptor status** |  |  |  |  |
| RNA expression low | 156 | 55.7 | 78 | 38.8 |
| RNA expression high | 124 | 43.3 | 123 | 61.2 |
| **Hormone receptor status**^1^ |  |  |  |  |
| RNA expression low | 78 | 27.9 | - | - |
| RNA expression high | 202 | 72.1 | - | - |
| **HER2 status** |  |  |  |  |
| RNA expression low | 245 | 87.5 | 186 | 92.5 |
| RNA expression high | 35 | 12.5 | 15 | 7.5 |
| **Metastasis** |  |  |  |  |
| Yes | 72 | 25.7 | 48 | 76.1 |
| No | 208 | 74.3 | 153 | 23.9 |

^1^The hormone receptor status is defined as positive when one of either the estrogen or the progesterone receptor status, is positive.

**S1 Table F:** Clinicopathological characteristics of node-negative breast cancer patients (fresh frozen tissue) from the Transbig cohort who did not develop a metastasis in the first three years after surgery (n=240).

|  | **All patients** | | **ER positive** | |
| --- | --- | --- | --- | --- |
| Characteristics | **n** | **%** | **n** | **%** |
| **Age at diagnosis** |  |  |  |  |
| <50 | 132 | 55.0 | 96 | 53.9 |
| ≥50 | 108 | 45.0 | 82 | 46.1 |
| **pT stage** |  |  |  |  |
| ≤2cm | 131 | 54.6 | 104 | 58.4 |
| >2cm | 109 | 45.4 | 74 | 41.6 |
| **Histological grade** |  |  |  |  |
| G I | 54 | 22.5 | 51 | 28.7 |
| G II | 94 | 39.2 | 82 | 46.1 |
| G III | 79 | 32.9 | 34 | 19.1 |
| Not documented | 13 | 5.4 | 11 | 6.2 |
| **Estrogen receptor status** |  |  |  |  |
| RNA expression low | 62 | 25.8 | - | - |
| RNA expression high | 178 | 74.2 | - | - |
| **Progesterone receptor status** |  |  |  |  |
| RNA expression low | 131 | 54.6 | 69 | 38.8 |
| RNA expression high | 109 | 45.4 | 109 | 61.2 |
| **Hormone receptor status**^1^ |  |  |  |  |
| RNA expression low | 62 | 25.8 | - | - |
| RNA expression high | 178 | 74.2 | - | - |
| **HER2 status** |  |  |  |  |
| RNA expression low | 211 | 87.9 | 166 | 93.3 |
| RNA expression high | 29 | 12.1 | 12 | 6.7 |
| **Metastasis** |  |  |  |  |
| Yes | 49 | 20.6 | 39 | 21.9 |
| No | 191 | 79.4 | 139 | 78.1 |

^1^The hormone receptor status is defined as positive when one of either the estrogen or the progesterone receptor status, is positive.
