# Supplementary material for: Epsin Family Member 3 and Ribosome-Related Genes Are Associated with Late Metastasis in Estrogen Receptor-Positive Breast Cancer and Long-Term Survival in Non-Small Cell Lung Cancer Using a Genome-Wide Identification and Validation Strategy
Source: PLoS One. 2016 Dec 7;11(12):e0167585. doi: 10.1371/journal.pone.0167585 (PMC5142791; doi:10.1371/journal.pone.0167585)
Supplement: S3 Table — (DOCX) [file pone.0167585.s007.docx]

**S3 Table:** Univariate analysis of the late-type probe sets in the period beyond five years after surgery in the validation cohort (Mainz). HR: hazard ratio; p: p-value.

| Affy ID | Gene symbol | HR | p |
| --- | --- | --- | --- |
| 200081_s_at | RPS6 | 0.17 | 0.089 |
| 200715_x_at | RPL13A | 0.10 | 0.003 |
| 200725_x_at | RPL10 | 0.02 | 0.006 |
| 200858_s_at | RPS8 | 0.05 | 0.016 |
| 200937_s_at | RPL5 | 0.15 | 0.011 |
| 205542_at | STEAP1 | 0.53 | 0.024 |
| 209134_s_at | RPS6 | 0.04 | 0.028 |
| 211073_x_at | RPL3 | 0.03 | 0.019 |
| 211938_at | EIF4B | 0.10 | 0.008 |
| 215963_x_at | RPL3 | 0.03 | 0.004 |
| 217877_s_at | GPBP1L1 | 0.03 | 0.002 |
| 220318_at | EPN3 | 2.90 | 0.006 |
