# Supplementary material for: Epsin Family Member 3 and Ribosome-Related Genes Are Associated with Late Metastasis in Estrogen Receptor-Positive Breast Cancer and Long-Term Survival in Non-Small Cell Lung Cancer Using a Genome-Wide Identification and Validation Strategy
Source: PLoS One. 2016 Dec 7;11(12):e0167585. doi: 10.1371/journal.pone.0167585 (PMC5142791; doi:10.1371/journal.pone.0167585)
Supplement: S8 Table — (DOCX) [file pone.0167585.s012.docx]

**S8 Table:** Validated late-type genes that predict metastasis-free survival three years after primary treatment and later in the three node-negative, untreated breast cancer cohorts and in a meta-analysis of all three cohorts (A). HR: hazard ratio; p: p-value; p (fdr): p-value after false discovery rate correction.

| **Affymetrix ID** | **Gene symbol** | **Rotterdam cohort** | | **Transbig**  **cohort** | | **Mainz**  **cohort** | | | **Meta-analysis**  **of all three cohorts** | | |
| --- | --- | --- | --- | --- | --- | --- | --- | --- | --- | --- | --- |
|  |  | **HR** | **p** | **HR** | **p** | **HR** | **p** | **p (fdr)** | **HR** | **p** | **p (fdr)** |
| **200081_s_at** | RPS6 | 0.32 | 0.010 | 0.30 | 0.017 | 0.09 | 0.005 | 0.037 | 0.27 | <0.001 | 0.029 |
| **200715_x_at** | RPL13A | 0.20 | 0.011 | 0.38 | 0.035 | 0.15 | 0.003 | 0.037 | 0.26 | <0.001 | 0.034 |
| **200725_x_at** | RPL10 | 0.08 | 0.003 | 0.09 | 0.026 | 0.03 | 0.007 | 0.044 | 0.07 | <0.001 | 0.016 |
| **200858_s_at** | RPS8 | 0.17 | 0.004 | 0.17 | 0.004 | 0.07 | 0.007 | 0.044 | 0.15 | <0.001 | 0.007 |
| **200937_s_at** | RPL5 | 0.34 | 0.012 | 0.28 | 0.001 | 0.15 | 0.003 | 0.037 | 0.27 | <0.001 | 0.005 |
| **205542_at** | STEAP1 | 0.74 | 0.046 | 0.68 | 0.013 | 0.51 | 0.004 | 0.037 | 0.67 | <0.001 | 0.043 |
| **209134_s_at** | RPS6 | 0.27 | 0.045 | 0.11 | 0.009 | 0.03 | 0.005 | 0.037 | 0.13 | <0.001 | 0.127 |
| **211073_x_at** | RPL3 | 0.10 | 0.001 | 0.19 | 0.046 | 0.02 | 0.002 | 0.037 | 0.10 | <0.001 | 0.008 |
| **211938_at** | EIF4B | 0.26 | 0.007 | 0.25 | 0.002 | 0.13 | 0.003 | 0.037 | 0.22 | <0.001 | 0.005 |
| **215963_x_at** | RPL3 | 0.10 | 0.001 | 0.17 | 0.019 | 0.04 | 0.001 | 0.037 | 0.10 | <0.001 | 0.005 |
| **217877_s_at** | GPBP1L1 | 0.31 | 0.029 | 0.24 | 0.023 | 0.03 | <0.001 | 0.030 | 0.17 | 0.002 | 0.245 |
| **220318_at** | EPN3 | 2.31 | 0.036 | 2.07 | 0.024 | 2.56 | 0.005 | 0.037 | 2.30 | <0.001 | 0.038 |
